# Interplay between arginine methylation and ubiquitylation regulates KLF4-mediated genome stability and carcinogenesis

**DOI:** 10.1038/ncomms9419

**Published:** 2015-09-30

**Authors:** Dong Hu, Mert Gur, Zhuan Zhou, Armin Gamper, Mien-Chie Hung, Naoya Fujita, Li Lan, Ivet Bahar, Yong Wan

**Affiliations:** 1 Department of Cell Biology, University of Pittsburgh School of Medicine, Pittsburgh, Pennsylvania 15213, USA; 2 University of Pittsburgh Cancer Institute, Pittsburgh, Pennsylvania 15213, USA; 3 Department of Computational and Systems Biology, University of Pittsburgh School of Medicine, Pennsylvania 15213, USA; 4 Department of Molecular and Cellular Oncology, The University of Texas MD Anderson Cancer Center, Houston, Texas 77030, USA; 5 Center for Molecular Medicine and Graduate Institute of Cancer Biology, China Medical University, Taichung 402, Taiwan; 6 Cancer Chemotherapy Center, Japanese Foundation for Cancer Research, Tokyo 135-8550, Japan; 7 Department of Microbiology and Molecular Genetics, University of Pittsburgh School of Medicine, Pennsylvania 15213, USA

## Abstract

KLF4 is an important regulator of cell-fate decision, including DNA damage response and apoptosis. We identify a novel interplay between protein modifications in regulating KLF4 function. Here we show that arginine methylation of KLF4 by PRMT5 inhibits KLF4 ubiquitylation by VHL and thereby reduces KLF4 turnover, resulting in the elevation of KLF4 protein levels concomitant with increased transcription of KLF4-dependent p21 and reduced expression of KLF4-repressed Bax. Structure-based modelling and simulations provide insight into the molecular mechanisms of KLF4 recognition and catalysis by PRMT5. Following genotoxic stress, disruption of PRMT5-mediated KLF4 methylation leads to abrogation of KLF4 accumulation, which, in turn, attenuates cell cycle arrest. Mutating KLF4 methylation sites suppresses breast tumour initiation and progression, and immunohistochemical stain shows increased levels of both KLF4 and PRMT5 in breast cancer tissues. Taken together, our results point to a critical role for aberrant KLF4 regulation by PRMT5 in genome stability and breast carcinogenesis.

## Abstract (web summary)

Krüppel-like factor 4 plays an important role in regulating responses to DNA damage, cell-fate decision and apoptosis. Here the authors show that aberrant regulation by methyltransferase PRMT5 results in failure to arrest the cell cycle and genome instability, pointing to a role in carcinogenesis.

## Graphical abstract

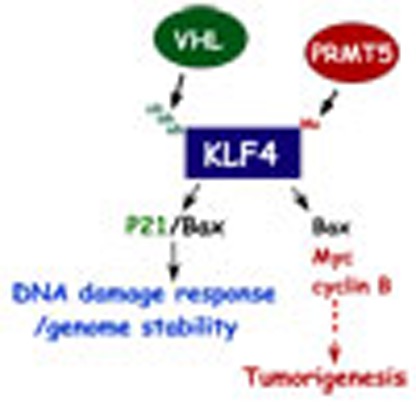

Krüppel-like factor 4 (KLF4, GKLF) is an important regulator of cell-fate decisions, including DNA damage response, inflammation, apoptosis and stem cell renewal^1^,^2^. Its impact on cancer formation has been recently indicated by the TCGA project (The Cancer Genome Atlas)^3^,^4^. As a transcription factor, KLF4 regulates various biological functions and tumorigenesis by activating or inhibiting a network of genes involved in cellular processes including cell-cycle control, genome stability, stem cell renewal, adhesion, apoptosis and metabolism^5^. Surprisingly, recent studies have sketched an ambivalent nature for KLF4 in tumorigenesis as either a tissue-specific tumour suppressor or an oncogene with the underlying mechanism remaining unclear^1^,^2^. KLF4 has been reported to have tumour-suppressive properties in gastrointestinal, oesophageal, lung and pancreatic cancer^6^,^7^, while it acts as an oncogenic factor in breast and squamous cell carcinoma^8^,^9^,^10^,^11^,^12^,^13^. Although KLF4 and its several downstream targets have been well dissected, especially in gastrointestinal and pancreatic cancer, it remains unclear why elevated KLF4 protein levels enhance malignant transformation in the mammary glands and skin^1^,^7^. Particularly, KLF4 regulation in response to a variety of environmental factors such as DNA damage lacks investigation^1^,^2^,^7^. On the basis of the observation that KLF4 is unstable and its protein half-life is remarkably altered in response to oncogenic signalling as well as various stress factors^14^,^15^, we focused on the identification of proteins that regulate KLF4 post-translationally, and here we report the functional interaction of KLF4 with PRMT5.

PRMT5 is a mammalian protein arginine methyltransferase that catalyses the addition of methyl groups to the guanidine nitrogen atoms of arginine^16^,^17^. Post-translational modification of proteins through arginine methylation usually alters their activity and the interactive property with other substrate proteins. Besides histone, the list of PRMT5-targeted regulatory proteins has recently expanded with the elucidation of its impact on a variety of cellular processes, including transcriptional regulation, DNA damage response/DNA repair, RNA metabolism as well as signalling modulation^16^,^17^,^18^. PRMT5 was initially linked to development and cellular proliferation in a mouse transgenic study, where the targeted deletion of *PRMT5* resulted in early embryonic lethality and suppression of pluripotency in ES cells by reprogramming a set of genes that orchestrate stem cell self-renewal and differentiation^19^. Inactivation of *PRMT5* in worms leads to genome instability in response to ionizing irradiation^20^,^21^, whereas proteomic study in fruit fly has unveiled the role of PRMT5 in RNA metabolism through methylation of the Piwi protein^22^. PRMT5 has attracted strong attention for its clinical impact as related to tumorigenesis and anticancer therapeutics initially simply because of its remarkable accumulation in blood, breast, colon and stomach cancers that promotes cell survival in the face of DNA-damaging agents^23^. Moreover, several critical proteins in oncogenic and apoptotic pathways such as CUL4A/B, EGFR and E2F have been shown to be regulated by PRMT5-mediated methylation^24^,^25^,^26^. Previous studies have shown that KLF4 is tightly regulated by different types of post-translational modifications, including phosphorylation, acetylation, sumoylation and ubiquitylation^7^,^24^, while for the first time we find and report here its modification by arginine methylation as well as the physiological consequence of this particular post-translational modification. Identification of the mechanism by which KLF4 is regulated via PRMT5-mediated methylation will address a crucial knowledge gap for the role of KLF4 and PRMT5 in tumorigenesis, which could provide novel strategies for anticancer therapy.

We recently reported that KLF4 is a rapidly turned over protein with its half-life governed by VHL-VBC ubiquitin protein ligase^5^. In this study, we demonstrate that PRMT5 directly interacts with KLF4 and catalyses the methylation of arginine 374, 376 and 377 on KLF4. This post-translational modification is proposed to alter the protein (KLF4) conformation and dynamics, which in turn antagonizes VHL/VBC-mediated KLF4 ubiquitylation through the alteration of recognition of KLF4 by VHL/VBC E3 ligase, thereby stabilizing KLF4. In the presence of genotoxic stress, disruption of PRMT5-mediated KLF4 methylation results in the failure of DNA damage-induced KLF4 upregulation, which in turn attenuates p21-mediated DNA damage response. During the process of cancer development, abolishment of KLF4 methylation leads to upregulated Bax levels and reduced expression levels for a set of oncogenes such as cyclin D2, cyclin B1, Myc, EGF and IGF1 because of enhanced destruction of KLF4 that suppresses transformation of mammary gland epithelial cell and, accordingly, breast tumour progression. Indeed, we find that PRMT5 upregulation tightly associated with an increase in KLF4 levels is a common feature in mammary cancer. Our findings not only explain the newly reported tumorigenic role for KLF4 in breast carcinogenesis by the TCGA project, but they also provide the basis for future studies on how the precise expression level of KLF4 is orchestrated by coordination between ubiquitylation and methylation and how PRMT5 affects KLF4 function in other processes such as stem cell and cancer-initiating cell reprogramming.

## Results

### Association of PRMT5 with KLF4

**Figure 1:**
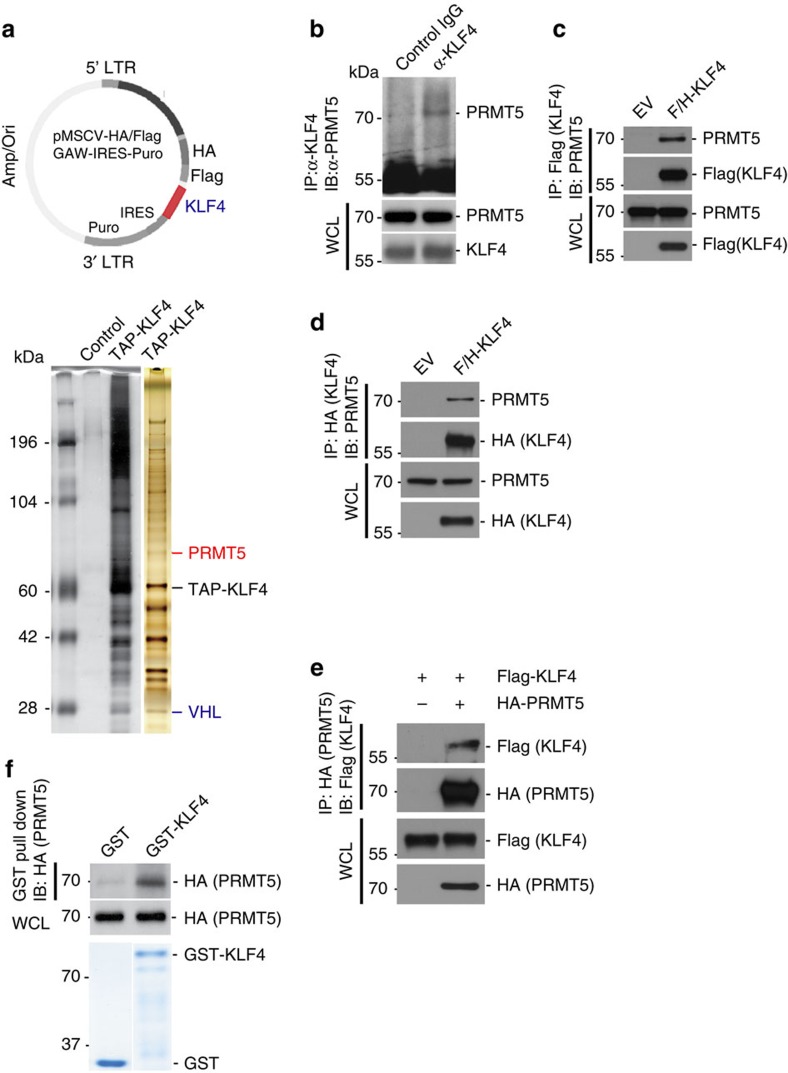
Identification of interaction between KLF4 with PRMT5. (**a**) Engineer of TAP-KLF4 stable clone and purification of the KLF4 protein complex. Proteins that interacted with KLF4 were purified from HeLaS3 cells stably expressing Flag and HA-tagged KLF4 or HeLaS3 (control). The nuclear extract was used for purification of KLF4 complex following by the standard TAP purification protocol. (**b**) Validation of PRMT5-KLF4 interaction *in vivo*. Endogenous interaction between KLF4 and PRMT5 was observed in HeLaS3 cell measured by immunoprecipitation. Normal IgG was used as the control. (**c**) Confirmation of interaction between KLF4 and PRMT5 using immunoprecipitation based on the TAP-KLF4 stable clone utilized for the purification of KLF4 complex in (**a**). (**d**,**e**) Validation of PRMT5-KLF4 interaction by immunoprecipitation based on expression and immunoprecipitation of KLF4 with different tag (HA). Cell lysates from HEK293T cells ectopically expressed the indicated plasmids were immunoprecipitated with the antibody again HA tag (IP: HA-KLF4/IB: PRMT5) or PRMT5 (IP: HA-PRMT5/IB: Flag-KLF4). (**f**) Direct interaction between PRMT5 and KLF4. Cell lysates from HEK293T cells transfected with HA-tagged PRMT5 were mixed with purified GST or GST-KLF4 immobilized on glutathione beads. Samples were electrophoresed and immuoblotted with the HA tag antibody. Levels of input protein are shown by immunoblotting with the HA tag antibody or Coomassie staining of SDS-PAGE gel.

We recently reported that KLF4 is a fast turnover protein with its half-life governed by the VHL ubiquitin protein ligase^5^. To search for other proteins that might regulate KLF4 proteolysis, we took a biochemical approach to identify further KLF4-interacting proteins using cells expressing tagged KLF4 to isolate KLF4 protein complexes by tandem immunopurification^5^,^27^. Our efforts led to the identification of several KLF4-binding partners, including PRMT5, by mass spectrometric analyses (Fig. 1a and Supplementary Fig. 1A). The interaction of PRMT5 with KLF4 was confirmed by blotting complexes with endogenous KLF4 precipitates with KLF4-specific antibodies (Fig. 1b and Supplementary Fig. 5A) or complexes of overexpressed tagged KLF4 precipitated with antibodies against two different tags (Fig. 1c,d) and probing for PRMT5. To rule out an artefact of recognition by the PRMT5 antibody, we also tested for the interaction of KLF4 with tagged PRMT5 using an antibody against the PRMT5 tag (Fig. 1e). Furthermore, we show that PRMT5 from lysates bound to bacterially purified KLF4 *in vitro* (Fig. 1f). Finally, we demonstrated that PRMT5 and KLF4 are colocalized using confocal microscopy (Supplementary Fig. 1B).

### Binding motifs and catalytic sites of KLF4 methylation

**Figure 2:**
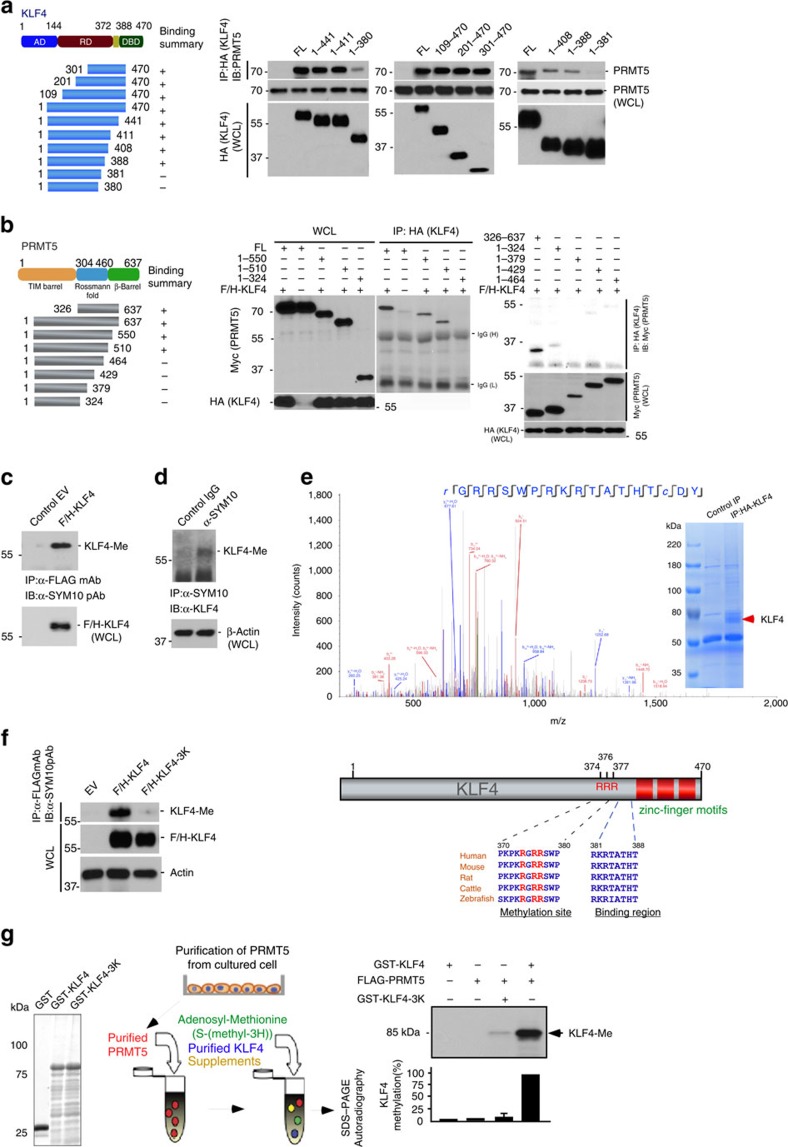
A carboxyl-terminal region of KLF4 and triple arginine sites on KLF4 mediate KLF4 methylation by PRMT5. (**a**) Mapping the molecular module on KLF4 interacting with PRMT5. KLF4 amino acids 382–388 is necessary for its binding to PRMT5. Flag and HA-tagged full-length or deletion mutants of KLF4 were immunoprecipitated with the HA antibody from transfected HEK293T cells and then blotted with PRMT5 antibody. AD, activation domain; RD, repression domain; NLS, nuclear localization signal; DBD, DNA binding domain (**b**) Identification of molecular region on PRMT5 interacting with KLF4. Amino acids 465–510 on PRMT5 is necessary for its binding to KLF4. (**c**) Ectopic KLF4 is methylated *in vivo*. Flag and HA-tagged KLF4 were immunoprecipitated with the Flag antibody from transfected HEK293T cells and then immunoblotted with the antibody SYM10 (The SYM10 antibody specifically recognizes protein containing symmetrically dimethylated arginine). (**d**) Endogenous KLF4 is methylated. Cell lysates from HeLaS3 cells were immunoprecipitated with the SYM10 antibody and then immunoblotted with the KLF4 antibody. Normal IgG was used as the control. (**e**) Identification of residues on KLF4 that mediate KLF4 methylation by mass spectrometry analysis. Flag and HA-tagged KLF4 were immunoprecipitated with the HA antibody from HeLaS3 cells, separated on SDS-PAGE and further subjected to mass spectrometry analysis. (**f**) Left panel, validation of arginine 374, 376 and 377 on KLF4 (RGRR methylation motifs) in mediating PRMT5-catalyzed KLF4 methylation. Right panel, amino acid sequence alignment of KLF4 protein from different species is represented. Three methylated arginine residues are highlighted. (**g**) Assay f*or* methylation of KLF4 by PRMT5 *in vitro* using purified proteins. Recapitulation of KLF4 methylation by PRMT5 *in vitro*. Left panel, coomassie blue staining of GST-fused KLF4 wide-type or mutant purified from *E.coli*. Middle panel, schematic representation of *in vitro* methylation assay. Right panel, Flag-tagged PRMT5 was purified from transfected HEK293T cells by immunoprecipitation using Flag antibody and incubated with purified GST-KLF4, or GST-KLF4 mutant. The reaction mixes were separated by SDS-PAGE and analyzed by autoradiography. The density of methylated KLF4 band was quantified. (Error bars indicate s.e.m. Data represent mean values of three independent experiments).

KLF4 functions as a versatile transcription factor that up- or downregulates its responsive genes through an activation domain or a repressive domain located near its amino terminus^1^,^2^,^7^. The carboxyl terminus of KLF4 contains three zinc-fingers (Supplementary Fig. 2A) and two nuclear localization signals that mediate DNA binding and regulate subcellular localization^1^,^2^,^7^. Previous gene structure and function studies have sketched several critical functional domains on PRMT5, including TIM-barrel or β-barrel domains at its amino- and carboxyl termini and a Rossmann fold in its central region^28^. To identify the regions on KLF4 as well as the motifs of PRMT5 that facilitate their interaction, we generated a series of Flag-HA-tagged deletion mutants for KLF4 and several Myc-tagged deletion mutants for PRMT5 and co-transfected them (Fig. 2a,b). Immunoprecipitates of HA-tagged KLF4 were tested for the presence of myc-tagged PRMT5. As shown in Fig. 2a, the interaction assays suggest that amino acids from K382-T388 of KLF4 and P465-H510 of PRMT5 are critical to mediate KLF4 interaction with PRMT5 (Fig. 2b). However, those assays did not provide us with the exact identity/location of the specific amino acids; we merely obtained information on the particular regions involved in KLF4-PRMT5 interaction. To identify the specific residues and explore their interactions, we performed extensive Molecular Dynamic simulations as described in the next section.

We next tested whether the arginine methyltransferase PRMT5 could methylate KLF4. To check for KLF4 arginine methylation, we tested precipitates of tagged KLF4 from cells with an antibody against symmetric dimethylated arginine (SYM10). KLF4, but not a control precipitate showed a signal at the expected protein size (Fig. 2c). We confirmed the methylation of KLF4 by testing precipitates with SYM10 for KLF4 (Fig. 2d). To identify KLF4 arginine sites that are methylated, we expressed HA-tagged KLF4 in HEK293 cells and purified KLF4 protein by immunoprecipitation. Empty vector served as control. The prominent band was excised and subjected to mass spectrometric analysis leading to the identification of R374 as methylation site (Fig. 2e). This site is in agreement with a conserved RGRR substrate motif reported for PRMT5 (ref. 29). To verify the importance of R374 for KLF4 methylation, we expressed point mutants of tagged KLF4 and tested immunoprecipitates for methylation. We observed that mutation R374K leads to substantial, but incomplete reduction of KLF4 methylation (Supplementary Fig. 9). In contrast, mutation of all three arginines (374, 376 and 377) in the PRMT5 substrate motif led to the complete inhibition of KLF4 methylation (Fig. 2f). To further determine whether KLF4 is a substrate for PRMT5, we recapitulated the KLF4 methylation i*n vitro* using purified proteins^24^,^25^,^26^. Bacterially purified GST–KLF4 fusion protein and PRMT5 purified from HeLa cells were incubated with S-(methyl-3H) adenosyl-methionine and supplements. Methylation of KLF4 was measured using autoradiography^24^,^25^,^26^. As shown in Fig. 2g, KLF4 methylation *in vitro* required PRMT5 and KLF4 arginines 374, 376 and 377.

### Structure and dynamics of the PRMT5-KLF4 complex

The above interaction and enzymatic assays suggest a direct interaction between KLF4 and PRMT5, which is facilitated by residues lying in the regions K382-T388 of KLF4 and P465-H510 of PRMT5 (Fig. 2a,b). Our structural modelling showed that these KLF4 and PRMT5 residue stretches are spatially located in the close neighbourhood of the methylation site of KLF4 (R374, R376 and R377) and of the active site of PRMT5 (E435 and E444), respectively. To further explore the interaction mechanism that facilitates KLF4 methylation by PRMT5 from a structural and dynamical point of view, we have conducted a total of 2 μs of full atomic MD simulation in explicit solvent (TIP3P (ref. 1) water and ions). All MD simulations were performed with NAMD 2.8 package^30^ and CHARMM27 force field^31^ using standard procedures (see MD Simulation Details in the SI-Supplementary Methods I).

KLFs contain three highly conserved zinc-finger motifs (Zf1–Zf3; Supplementary Fig. 2A) that bind to the GC/GT-rich regions of DNA^32^,^33^. Our experimentally identified interaction region is adjacent to the N-terminal end of Zf1 (Supplementary Fig. 2B,C). Structural data for this region are missing in all available KLF4 crystal structures^34^,^35^ presumably due to the high conformational variability of this region. Moreover, the methylation site (R374, R376 and R377) is not resolved either in known crystal structures. We selected the PDB structure 2WBS for our KLF4 simulations and, as a first step, modelled the missing stretch of amino acids, PKPKRGRRSWPRKR (residues 370–383; See Modelling the N-terminal region of the unbound KLF4 section in SI).

The human PRMT5 dimer structure for the simulations was taken from its hetero-octameric complex with MEP50 (PDB ID: 4GQB (ref. 18; See Modeling PRMT5 Structure section in SI). Residues P465-H510 are located at the bottom of the large cavity formed by the PRMT5 dimer (Supplementary Fig. 2D,E), comprising the dimerization domains. The arginine side chain of the substrate was proposed to access the *active* site on insertion through a narrow tunnel created by residues L312, F327 and W579, where it forms salt bridges with highly conserved E435 and E444 (Supplementary Fig. 2F)^18^, which permitted us to adopt a starting conformer for the three arginines of the KLF4 methylation site, for refinement by stimulation. The PRMT5-active site is not large enough to simultaneously accommodate all of the three arginines at once, strongly suggesting that those arginines get methylated one at a time.

We refined the binding mode between KLF4 and PRMT5 by a five-step MD simulation protocol that incorporated all of the above-listed experimental data (See Modelling KLF4-PRMT5-binding section in SI); (i) first KLF4 was steered into the large cavity formed by the PRMT5 dimer so that the experimentally detected binding regions were in close proximity; (ii) then R377 was steered into the active site through the narrow tunnel formed by L312, F327 and W579, to tightly pin down KLF4 on PRMT5; (iii) by keeping R377 at the active site, an initial bound conformation was generated on the basis of the surface chemical properties of the binding regions (Supplementary Fig. 2G and Supplementary Movie 1). R377 was removed from the active site and subsequently R374 was steered into the active site consistent with mass spectrometry data; (iv) subsequently, the binding pause was refined with the help of a series of MD runs performed for KLF4 substructures (Supplementary Fig. 2H,I); (v) full KLF4 conformations were generated and further refined by performing long MD simulations (Supplementary Fig. 3). Computations were repeated twice to verify the close reproducibility of the resulting refined structure for the KLF4–PRMT5 complex. Below we present results from the first set of computations; the second is discussed in the The PRMT5–KLF4 complex from the second run section in SI.

**Figure 3:**
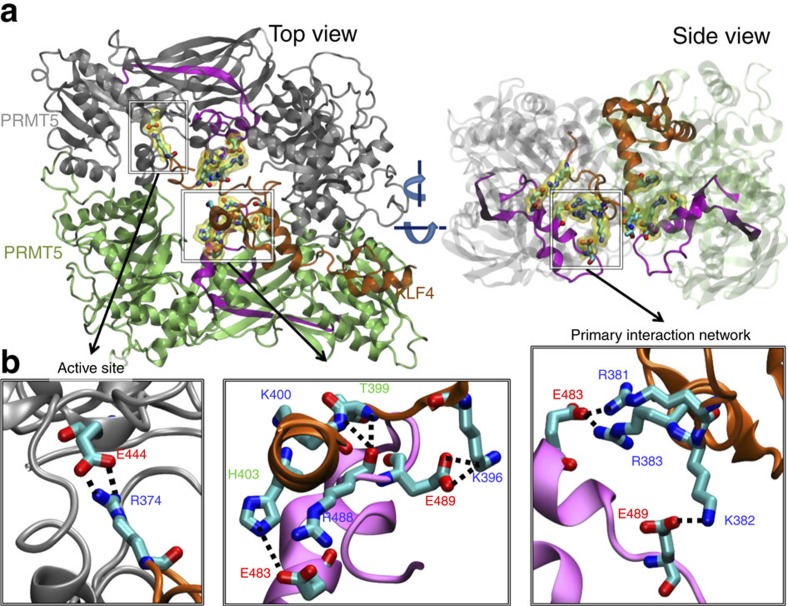
PRMT5-KLF4 complex and interaction between them. (**a**) *Top* and *side* views of the complex obtained from the first run (after 230 ns MD simulation) is shown at the upper part. PRMT5s are shown in grey and lime. The experimentally predicted interaction region on PRMT5 is shown in magenta. KLF4 is shown in *orange*. In lower panel (**b**) on the left the interaction between KLF4 residue R374 and PRMT5 residue E444 at the active site is shown. In the lower middle panel interactions between Zf1 (excluding the experimentally predicted binding region) and the experimentally predicted PRMT5 binding region are shown. On the lower right panel the primary interaction network, which is closely located to the methylation sites of KLF4, between the experimentally predicted binding regions is shown. Interacting residues are shown in licorice representation and colored according to their atom types. In panel (**a**), these residues are further highlighted with transparent yellow surface representations.

As explained above, R374 has been originally positioned in the vicinity of both E435 and E444 at the active site of PRMT5. However, as the simulations evolved, the interaction with E444 became prevalent. A strong interaction network comprising the positively charged residues R381, K382, R383 on KLF4 and negatively charged residues E483, E489 on PRMT5 became prominent. This interaction network is shown in Fig. 3 and Supplementary Fig. 4 for the first and second sets of simulations, respectively. In the first set, salt bridges formed between E483 (PRMT5) and KLF4 residues R381 and R383, and between KLF4 K382 and PRMT5 E489. These results suggest that the highly charged dimerization domain of PRMT5 acts like an anchor stabilizing the complex. This primary network was complemented by interactions involving other Zf1 residues and the region P465-H510 of the adjacent PRMT5 protomer (shown in lime in the figures). Strikingly, in parallel to the first subunit of the PRMT5 dimer, residues E483 and E489 of the second subunit also formed strong interactions with KLF4 (salt bridges E483-H403 (KLF4) and E489-K396 (KLF4)). These interactions are further consolidated by hydrogen bonds between backbone amines and carbonyls. The fact that two independently performed simulations provide almost identical binding characteristics strongly supports the binding modes and the corresponding key residues that we have identified.

Taken together, the above MD results highlight the significance of the charged residue stretch RKR (381–383) adjacent to the methylation sites of KLF4. Both PRMT5 subunits of the dimer are involved in the KLF4 binding, while one of them (the first) is juxtaposed to catalyse the methylation of KLF4 arginines. This particular subunit is engaged in tight interactions with KLF4, whereas the second seems to stabilize Zf1 in a proper orientation. In our simulations, Zf2 and Zf3 of KLF4 bound to the outer surface of the PRMT5 dimer, potentially giving them the freedom to bind DNA.

### Mechanistic role of protein methylation in regulating KLF4

**Figure 4:**
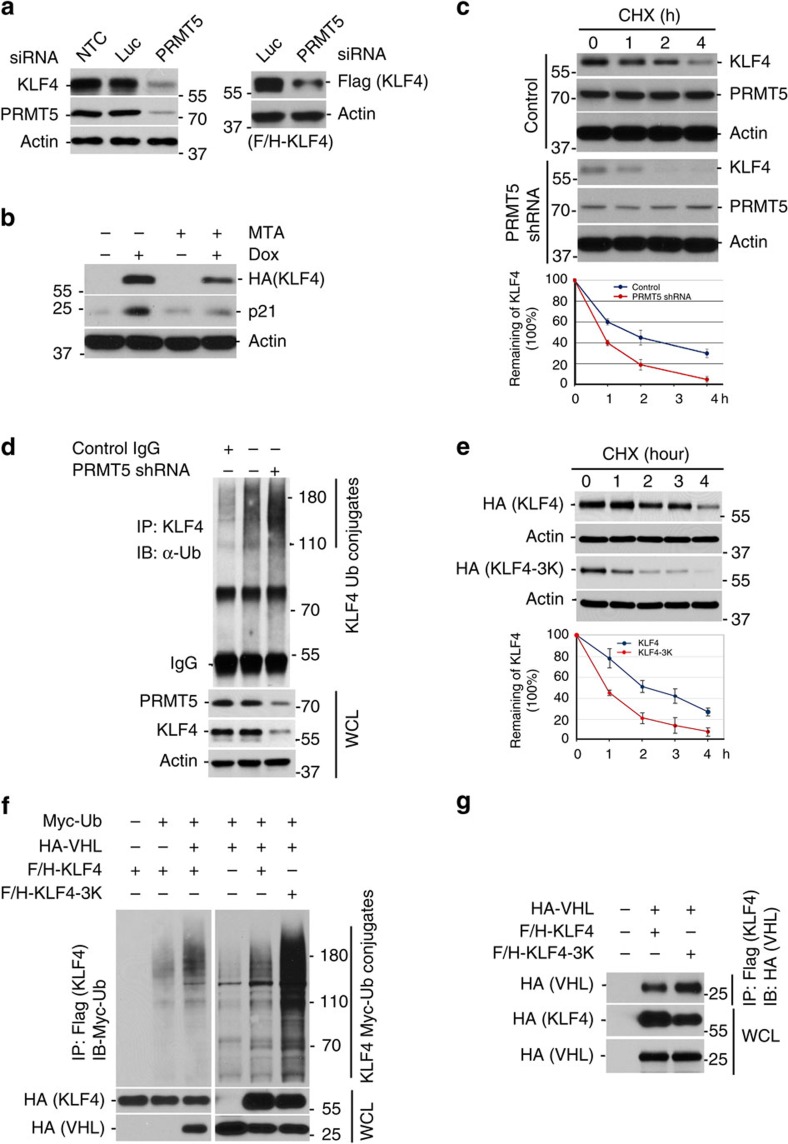
KLF4 protein stability is orchestrated by the crosstalk between PRMT5 and VHL mediated post-translational modification. (**a**) PRMT5 depletion leads to KLF4 protein downregulation. Left panel, HCT116 cells were transfected with PRMT5 siRNA or control luciferase siRNA. The expression of either endogenous (left panel) or ectopically expressed Flag-tagged KLF4 (right panel) was detected by immunoblotting. (**b**) MTA, a PRMT5 inhibitor, enhances the drop of KLF4 protein levels. Flag and HA-tagged KLF4 inducible U2OS cells were treated with 10 nM of doxycycline and 300 μM of 5'-methylthioadenosine (MTA). Cell lysates were immunoblotted with the antibody against HA. (**c**) PRMT5 depletion significantly promotes KLF4 protein turnover. HCT116 cells were transfected with PRMT5 shRNA or scramble (batch clones) following by the treatment with 20 μM cycloheximide. Cells were collected at the indicated time for immunoblotting using antibodies against KLF4 and PRMT5. The density of KLF4 band was quantified, normalized to the internal control β-actin and plotted. (**d**) PRMT5 depletion leads to an increased KLF4 ubiquitin conjugates. PRMT5 was depleted in HCT116 cells. Cell lysates were immunoprecipitated with the KLF4 antibody and then immunoblotted with the antibody against ubiquitin. (**e**) Disruption of KLF4 methylation results in fast turnover of KLF4 protein. Flag and HA-tagged KLF4 wide-type or mutant was transfected to HCT116 cells. Cells were treated with cycloheximide and collected at the indicated time for immunoblotting with the HA tag antibody. The density of KLF4 band was quantified, normalized to the internal control β-actin and presented. (**f**) Disruption of KLF4 methylation significantly enhances the VHL-mediated KLF4 ubiquitylation. HEK293T cells transfected with the indicated plasmids were treated with 10 μM MG132 for 6 h and collected for immunoprecipitation assay with the Flag tag antibody followed by the immunoblotting analysis with the Myc tag antibody. (**g**) Attenuation of KLF4 methylation enhances its affinity binding to VHL. HEK293T cells were transfected with the indicated plasmids and subjected to the same treatment as (**f**). Cell lysates were immunoprecipitated with the Flag tag antibody followed by the immunoblotting with the HA tag antibody. (Error bars from panel C&E indicate s.e.m. Data represent mean values of three independent experiments)

Methylation by PRMT5 has been reported to regulate proteins that orchestrate various cellular processes, including cell-cycle control, signal transduction, DNA damage response and DNA repair, immune response activation and tumorigenesis^16^,^17^,^24^,^25^,^29^,^36^. To analyse how PRMT5-mediated methylation affects KLF4, we knocked down PRMT5 in HCT116 cells as well as U2OS and MDA-MB-231 cells and measured the alteration of KLF4 abundance by immunoblotting. We observed that depletion of PRMT5 resulted in a significant drop of KLF4 protein levels (Fig. 4a and Supplementary Fig. 5B,C). Consistent with the importance of PRMT5 activity for KLF4 regulation, the PRMT5 inhibitor MTA causes a decrease in cellular KLF4 levels (Fig. 4b). To determine whether PRMT5 affects KLF4 protein turnover and whether the PRMT5-mediated alteration of KLF4 protein abundance involves ubiquitin-dependent proteolysis of KLF4, we measured KLF4 protein half-life as well as KLF4 ubiquitylation under normal or PRMT5 knockdown condition. As shown in Fig. 4c,d, depletion of PRMT5 leads to enhanced KLF4 protein turnover and increased KLF4 ubiquitylation. In addition, mutation of KLF4 methylation sites (replacement of arginines 374, 376 and 377 by lysines) results in accelerated KLF4 fast turnover (Fig. 4e).

The above results in Fig. 4a–e suggest a role for PRMT5 in modulating ubiquitin-dependent KLF4 turnover. Given our previous finding that VHL/VBC ubiquitylates KLF4 (ref. 5), we asked whether PRMT5-mediated methylation counteracts or suppresses KLF4 ubiquitylation by VHL/VBC. Consistent with the observed increased ubiquitylation of KLF4 after PRMT5 knockdown, mutation of PRMT5-specific methylation sites on KLF4 also leads to increased KLF4 ubiquitylation by VHL (Fig. 4f). This novel observation reveals a crosstalk between methylation and ubiquitylation in the orchestration of KLF4 protein stability. At this point, several possible mechanisms, such as change of KLF4 protein conformation, alteration of KLF4 recognition by its ubiquitin E3 ligase or interference of catalysis of ubiquitin-chain formation on KLF4, could mediate the methylation-facilitated alteration of KLF4 ubiquitylation. We therefore measured the affinity between E3 ligase VHL/VBC and wild-type KLF4 or methylation-resistant KLF4, respectively. As shown in Fig. 4g, mutation of methylation sites on KLF4 enhances the binding affinity between KLF4 and VHL, indicating that PRMT5-mediated methylation of KLF4 could affect the recognition of KLF4 by VHL, thereby lowering VHL-mediated KLF4 ubiquitylation. Collectively, our results indicate that KLF4 methylation by PRMT5 stabilizes KLF4 by antagonizing VHL-mediated KLF4 ubiquitylation.

### Regulation of KLF4 by PRMT5 ensures DNA damage response

**Figure 5:**
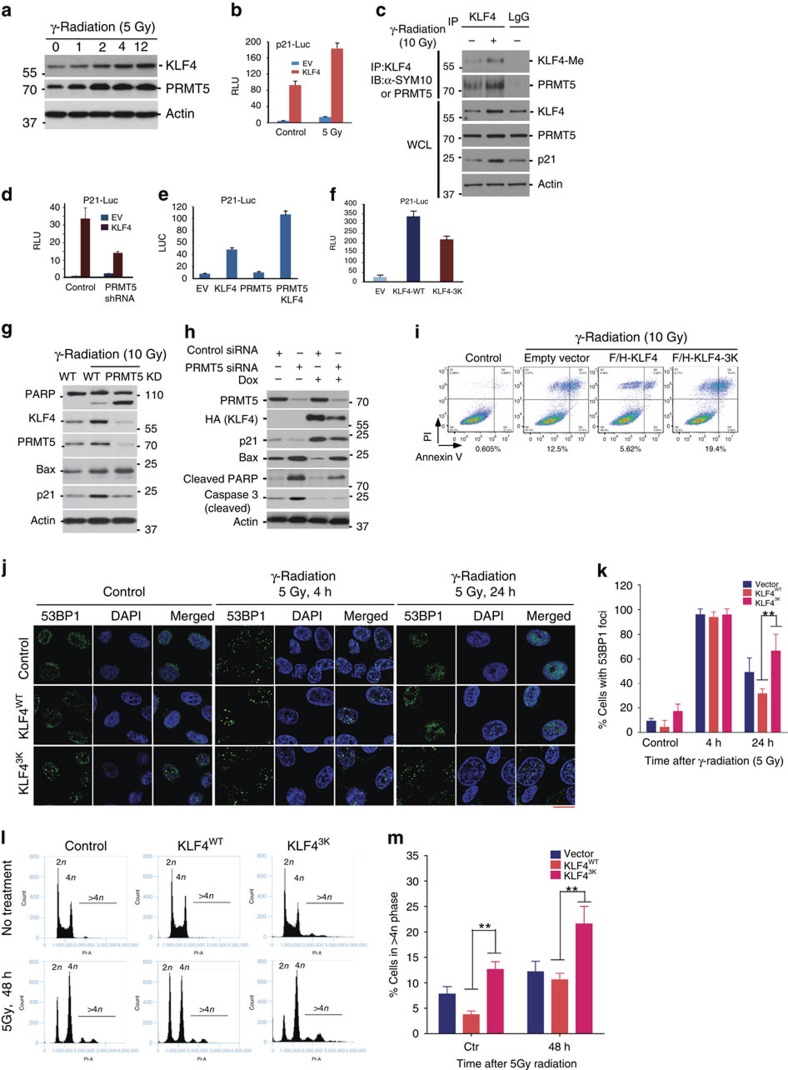
Interplay between PRMT5 and VHL determines cellular fate in response to DNA damage. (**a**) Both PRMT5 and KLF4 protein expression in U2OS cell are rapidly upregulated in response to γ-radiation (5 Gy). (**b**) Activation of p21 reporter is significantly enhanced by elevation of KLF4 in response to DNA damage signal. KLF4 was co-transfected with p21-Luc and an internal control *Renilla*-Luc. Transfected U2OS cells were treated with γ-radiation (5 Gy) and harvested for dual-luciferase assay. The firefly luciferase activity was normalized to *Renilla* and presented as RLU. (**c**) KLF4 methylation and its interaction with PRMT5 are significantly elevated in response to ionization. Cell lysates from U2OS cells treated with γ-radiation were immunoprecipitated with the KLF4 antibody and then immunoblotted with the indicated antibodies. (**d**) PRMT5 depletion attenuates the KLF4-mediated p21 transcription. PRMT5 siRNA was transfected into cells. 24 h after transfection, cells were transfected with p21-Luc for additional 24 h and then harvested for dual-luciferase assay. (**e**) Interplay between KLF4 and PRMT5 dramatically promotes activation of p21 reporter. (**f**) Methylation deficient mutant shows compromised ability to activate p21 reporter. (**g**) PRMT5 depletion leads to KLF4 downregulation that in turn induces the onset of apoptosis. PRMT5 siRNA was transfected into U2OS. 24 h after transfection, cells were treated with γ-radiation (10 Gy) and harvested for immunoblotting. (**h**,**i**) Induction of KLF4 expression antagonizes the apoptosis induced by PRMT5 depletion. (**h**) U2OS cells with stably inducible expression of KLF4 were transfected with PRMT5 siRNA. 24 h after transfection, cells were treated with doxycycline for 16 hours as well as γ-radiation (10 Gy) for 24 h and harvested for immunblotting. (**i**) Apoptotic analysis of U2OS, U2OS-KLF4-WT and U2OS-KLF4-3K cells in response to γ-radiation. Cells were treated with 10 Gy γ-radiation for 24 h and then the population of apoptotic cells was analyzed by using Annexin V-propidium iodide (PI) staining and flow cytometry. (**j**) While elevated KLF4 helps to remove the damaged DNA in U2OS cell after exposure to γ-radiation (5 Gy), interference of KLF4 protein stability by expression of KLF4 methylation-resistant mutant leads to retardation of removal of DNA damage lesions and genome instability. Formation of 53BP1 foci were observed and counted to reflect the DNA damage lesions. Scale bar, 10 μm. (**k**) Quantification of (**j**). (**l**) Interference of KLF4 methylation by expressing methylation-resistant KLF4 mutant leads to an increase of aneuploidy after ionization (5 Gy). The percentage of cells containing DNA content greater than 4 N was quantified. (**m**) Quantification of (**l**). (Error bars from panel (**b**,**d**–**f**,**k**,**m**) indicate s.e.m. Data represent mean values of triplicate cultures. **P<0.001 using Student's *t*-test.)

Both KLF4 and PRMT5 are thought to be critical in the maintenance of genome stability^29^,^37^,^38^. While targeted deletion of KLF4 leads to aberrant DNA damage checkpoint function as well as chromosomal instability, silencing PRMT5 results in abnormal DNA damage response and repair^20^,^29^,^37^,^39^,^40^,^41^. A threshold level of KLF4 is critical for determining cellular fate after cellular exposure to genotoxic stress. Transactivation of KLF4 leads to upregulation of p21 and downregulation of Bax, which are two pivotal elements in determining cell cycle arrest or onset of apoptosis^7^,^39^. After DNA damage, high KLF4 levels lead to elevated p21 protein abundance and ensure cell survival, while low KLF4 levels result in increased Bax levels that in turn trigger onset of cellular apoptosis^39^,^42^. To explore if the interplay between PRMT5 and KLF4 is important in the DNA damage response and how the methylation of KLF4 protein affects genome stability, we initially measured alterations in PRMT5 and KLF4 following DNA damage. As shown in Fig. 5a, both PRMT5 and KLF4 protein levels increase dramatically in response to ionizing radiation at a clinical dose. Gentoxic stress also increased p21 transcription in a KLF4 dependent manner, as measured by a luciferase based promoter assay (Fig. 5b). Concomitant with KLF4 accumulation after ionizing radiation, we observed an increase of KLF4 methylation as well as an enhanced interaction between KLF4 and PRMT5 (Fig. 5c).

To test the role of PRMT5 in KLF4-dependent p21 transcription, we measured p21 RNA levels after overexpression of KLF4 in the absence or presence of siRNA against PRMT5. Knockdown of PRMT5 leads to impaired KLF4 mediated p21 activation (Fig. 5d). PRMT5 overexpession on the other hand, enhanced KLF4-dependent p21 transcription (Fig. 5e). Mutation of KLF4 at its methylation sites impairs the activation potential of KLF4 for p21 (Fig. 5f and Supplementary Fig. 6A). Depletion of PRMT5 by RNA interference prior to ionizing radiation led to abolished DNA damage-induced KLF4 accumulation, resulting in decreased p21 expression and upregulation of Bax both in the case of endogenous KLF4 (Fig. 5g and Supplementary Fig. 6B) and overexpressed KLF4 (Fig. 5h). Knockdown of PRMT5 also increased PARP and Caspase 3 cleavage (Fig. 5h). Moreover we observe, by FACS analysis, that KLF4 overexpression has an antiapoptotic effect after ionizing radiation that is not seen if KLF4 is mutated at its methylation sites (Fig. 5i, Supplementary Fig. 6C,D and Supplementary Fig. 7A). In addition, while elevated KLF4 helps to remove the damaged DNA after exposure to γ-radiation (5 Gy), interference of KLF4 protein stability by expression of KLF4 methylation-resistant mutant leads to retardation of removal of DNA damage lesions (Fig. 5j,k). Besides the DNA damage and repair, we also measured the impact of KLF4 methylation in chromosomal stability. As shown in (Fig. 5l,m), results from the flow cytometry analysis indicate that interference of KLF4 methylation by expressing methylation-resistant KLF4 mutant leads to an increase of aneuploidy (DNA content >4 N) after exposure to ionization, suggesting a direct involvement of the PRMT5-mediated KLF4 methylation in the maintenance of genome stability^43^. Taken together these results suggest that the interplay between KLF4 and PRMT5 plays a critical role in mediating the DNA damage response. DNA damage-induced PRMT5 leads to increased methylation of KLF4 that in turn results in accumulation/stabilization of KLF4 through antagonizing its turnover. PRMT5-mediated accumulation of KLF4 is necessary to calibrate DNA damage induced upregulation of p21 and downregulation of Bax.

### Deregulated KLF4 methylation promotes tumorigenesis

**Figure 6:**
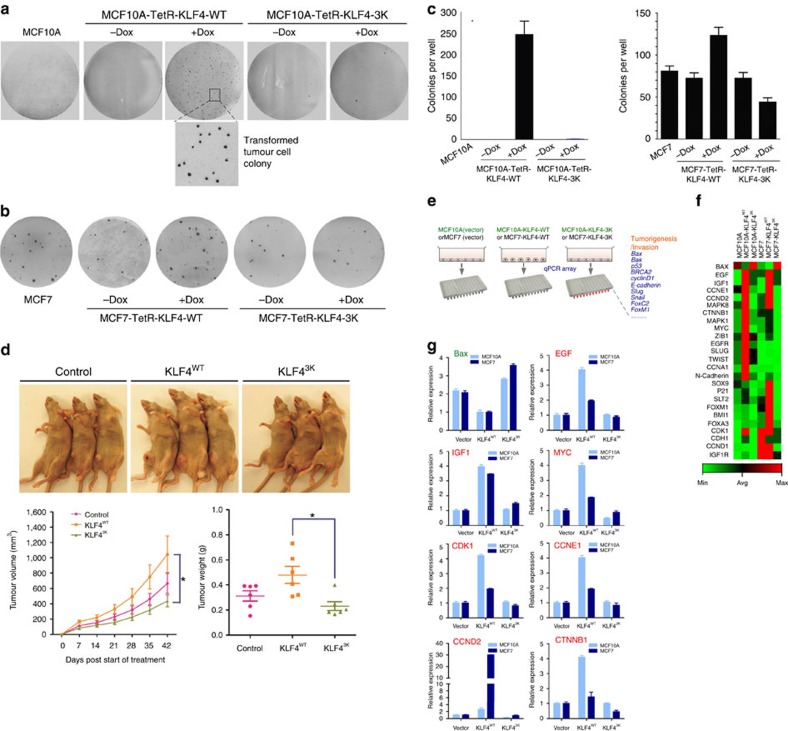
PRMT5-dependent methylation contributes to the oncogenic function of KLF4 in breast cancer initiation and invasion. (**a**) Colony formation assay on soft agar were done to evaluate the transformation of MCF10A, MCF10A-KLF4 and MCF10A-KLF4-3K cells. 2 × 10^4^ cells were plated on 35 mm dishes and cultured for 3 weeks. While elevated KLF4 expression promotes MCF10A transformation, expression of methylation-resistant KLF4 mutant fails to transform MCF10A. (**b**) While elevation of KLF4 enhanced tumor cell progression, expression of methylation-resistant KLF4 mutant failed promote the progression of breast tumor. Colony formation assay on soft agar were done to evaluate the transformation of MCF7, MCF7-KLF4 and MCF7-KLF4-3K cells. 4 × 10^3^ cells were plated on 35 mm dishes. Colonies were countered after 3 weeks. (**c**) Quantification of A and B. Error bars indicate s.e.m. Data represent mean values of triplicate cultures. (**d**) An *in vivo* mouse xenograft study was conducted to validate the oncogenic role of KLF4 methylation in breast tumor progression. MCF7 cells with stable expression of KLF4 or KLF4-3K were implanted into mammary fat pad of nude mice carrying estrogen pellets. Tumor volume was measured once a week and mice were sacrificed 42 days after injection. Upper panel, representative mice showing tumor formation. Lower panel, quantification for tumor growth curve based on the tumor volume (left panel) and tumor weight when mice were sacrificed (right panel). The results were presented as mean values±s.e.m. *P<0.01 using Student's *t*-test. (**e**) A schematic representation of customized breast cancer genes profile quantitative real-time PCR (qPCR) array. (**f**) Heat map representing gene expression changed in MCF10A, MCF10A-KLF4 and MCF10A-KLF4-3K as well as in MCF7, MCF7-KLF4 and MCF7-KLF4-3K cells. Values in the graph are expressed as mean±s.e.m. (**g**) Summary of validation of eight responsive genes in (**e**) using qPCR analyses.

Protein levels for both PRMT5 and KLF4 need to be tightly regulated, since aberrant expressions of PRMT5 or KLF4 have been correlated with poor prognosis in various types of tumors^1^,^2^,^16^. Recent observations by us and others have implicated KLF4 as potential tumor enhancer in mammary cancer^3^,^13^,^42^,^44^,^45^,^46^. A recent genome-wide association study indicates that aberration of three master genes, including KLF4, estrogen receptor and c-Myc, could be important causal factors for breast carcinogenesis^3^,^4^. To test whether deregulation of KLF4 protein stability by aberrant PRMT5 affects transformation of mammary gland epithelial cell or breast tumor progression, we engineered MCF10A and MCF7 cell lines with stable and inducible expression of KLF4 and KLF4-3K^42^. These cells were then seeded on soft agar for colony formation. As shown in Fig. 6a,c, induced expression of KLF4 by the presence of doxycycline enhances transformation of mammary gland epithelial cells, while expression of KLF4 with mutated methylation sites fails to achieve colony formation. In addition, suppression of KLF4 induced colony formation by mutating KLF4 methylation sites is observed in MCF7 cells (Fig. 6b,c). To validate the oncogenic role of arginine methylation of KLF4 in breast cancer, we further conducted an *in vivo* mouse xenograft study. As shown in Fig. 6d, in consistence with the observation in the cultured-cell model, elevated expression of KLF4 significantly promotes the breast tumor progression in comparison with the control group, while interference of KLF4 methylation by expression of the methylation-resistant KLF4 mutant fails to enhance the breast tumor progression^47^. In summary, the above-described findings indicate that regulation of KLF4 by PRMT5-mediated methylation is important for breast cancer development and show that mutating methylation sites of KLF4 and interference in the stability of KLF4 can affect tumor initiation and progression in breast cancer.

### Molecular basis for PRMT5-KLF4-mediated tumorigenesis

To dissect the possible molecular mechanisms how an aberrant interplay of PRMT5 and KLF4 contributes to breast tumor initiation and progression, we performed gene array assays and identified the downstream gene network responding to the aberrant KLF4 methylation by PRMT5 by using a customized breast cancer gene profile with quantitative real-time PCR (qPCR) array from SABiosciences (Fig. 6e)^48^,^49^. The expression of 111 breast carcinogenesis-associated genes with 6 control genes were measured and compared under three different conditions of KLF4 overexpression in both non-tumorigenic mammary gland epithelial cell line MCF10A and cancer cell line MCF7: overexpression of KLF4-WT; expression of methylation-resistant KLF4-3K or control vector. As demonstrated by cluster analysis displayed as a heat map^48^,^49^,^50^, we observed substantial alteration of expression in 25 genes in the breast cancer gene pool (Fig. 6f). As expected, a significant drop of Bax expression levels is observed under elevated expression of KLF4. Expression of methylation-resistant KLF4 (KLF4-3K) results in a profound increase of Bax levels in both MCF10A cells and MCF7 cells, indicating that interference of by the methylation resistant KLF4 mutant could antagonize the suppression of cell apoptosis caused by the elevation of KLF4 in breast carcinogenesis (Fig. 6f). We furthermore observed that a series of oncogenic signaling genes such as EGF, IGF1, EGFR, MAPK and cell cycle genes CCNE1, CCND2, CDK1 were markedly upregulated by elevated expression of KLF4 in both MCF10A cells and MCF7 cells, which is consistent with the previous reported role for KLF4 in promoting cell growth in mammary gland and breast tumor cells^42^. Moreover, we observed significant upregulation of several crucial stem cell factors such as Myc, Sox9 and BMI1 in response to elevated expression of KLF4 but not KLF4-3K, which suggests a critical role of KLF4 in the maintenance of self-renewal for breast cancer stem cell^7^. In addition, we observed increased expression of several metastatic genes in response to elevated wild type KLF4 but not KLF4-3K, including ZIB1, Slug, Twist, N-Cadherin. This is consistent with a previously described role of KLF4 in promoting breast tumor metastasis^51^,^52^. To validate the results obtained from the gene array, we carefully analyzed eight representative genes by using quantitative real-time PCR, including Bax, EGF, IGF1, MYC, CDK1, CCNE1, CCND2 and CTNNB1 in both MCF10A and MCF7. As shown in Fig. 6g, the alteration of expression of the eight genes is consistent with results obtained from the qPCR array. Taken together, the above results suggest that elevated KLF4 levels, such as caused by increased expression of PRMT5, could drive malignant transformation of mammary gland epithelial cell and further enhance breast tumor invasion through disruption of cellular regulation at multiple levels, such as apoptosis, cell growth, cell migration and cancer stem cell self-renewal.

### Deregulation of PRMT5 and KLF4 in breast cancer tissues

**Figure 7:**
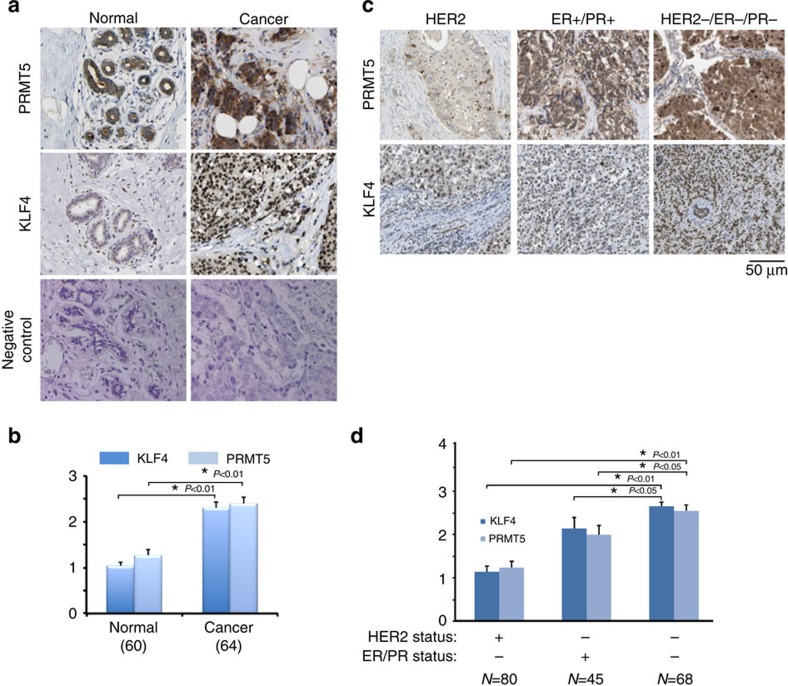
Aberrant accumulation of PRMT5 and KLF4 correlate with poor prognosis of breast cancer. (**a**,**b**). Accumulated KLF4 and PRMT5 protein was observed in human breast cancer tissue by IHC in comparison with adjacent normal breast tissue. (**a**) tissue arrays of 64 human breast cancer specimens with adjacent normal breast tissue were subjected to immunohistochemistry with anti-KLF4 and anti-PRMT5 antibodies and visualized by DAB staining. (**b**) Summary of (**a**). (**c**,**d**). Severe accumulation of KLF4 and PRMT5 protein in triple negative breast cancer tissue was detected in comparison with HER2 and ER/PR positive type breast tumors. Tissue arrays of 193 human invasive breast cancer specimens, including 80 HER2^+^, 45 ER/PR^+^ and 68 triple negative breast cancer tissues, were detected. The represented staining were shown in (**c**) and summarized in (**d**). The results were presented as mean values±s.e.m. *P<0.01, *P<0.05 using Student's *t*-test. Scale bar, 50 μm.

The above characterizations based on the cultured-cell model have demonstrated the biological consequence and physiological relevance of crosstalk regulation of KLF4 by ubiquitylation and protein methylation in the maintenance of genome stability as well as tumorigenesis. While our results suggest the importance of the interplay between KLF4 and PRMT5 in determining the cellular fate after the exposure to DNA damage signal, we observed that the aberrant KLF4 abundance due to the deregulated KLF4 regulation by PRMT5 could directly enhance the breast tumor initiation and progression. In order to further explore the possible clinical relevance of KLF4-PRMT5 axis in tumorigenesis, we decided to examine if disrupted regulation of KLF4 by PRMT5 is a driving factor for breast tumor initiation and progression. To this end, we measured the protein expression levels of PRMT5 and KLF4 in human breast tumor and adjacent normal tissue by immunohistochemistry (IHC). As shown in Fig. 7a,b, the protein levels of both PRMT5 and KLF4 are significantly higher in breast tumor tissues than that of in the adjacent normal tissues, where their expression are well correlated with the histological grade (Supplementary Tables 3 and 4). Interestingly, the observed accumulation of both PRMT5 and KLF4 is strongest in triple negative type breast cancer (see comparison with HER+ or ER+/PR+ type breast cancer in Fig. 7c,d). Thus, increased levels of PRMT5 and KLF4 are highest in tissues from breast cancers associated with poor prognosis.

## Discussion

**Figure 8:**
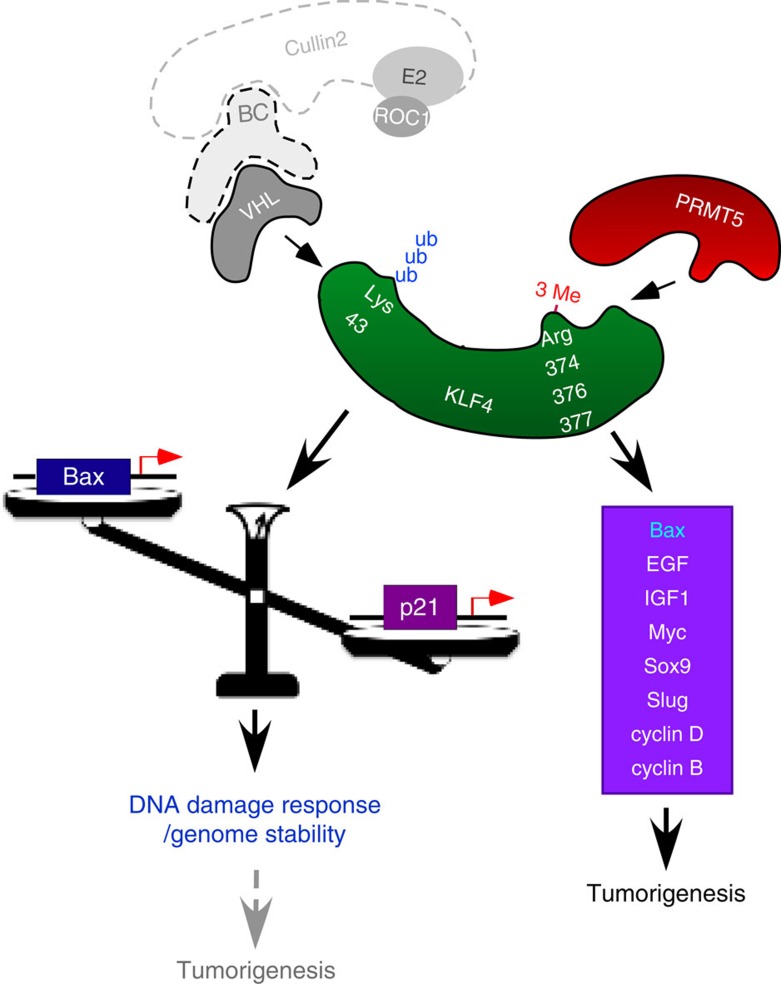
Interplay between PRMT5 and VHL on KLF4 regulates genome stability and tumorigenesis. KLF4 protein is coordinately regulated by PRMT5-dependent arginine methylation and VHL-dependent ubiquitylation. Following genotoxic stress, disruption of PRMT5-mediated KLF4 methylation leads to abrogation of KLF4 accumulation, which, in turn, attenuates cell cycle arrest. During the tumorigenesis, downregulation of Bax or upregulation of several oncogenes due to aberrant KLF4 accumulation results in mammary tumor initiation and progression.

We previously reported that VHL-mediated ubiquitination targets KLF4 for proteasomal degradation and thereby regulates the constitutive turnover of KLF4 (ref. 5). Here we report for the first time that KLF4 ubiquitination is regulated by the arginine methyltransferase PRMT5. Our molecular characterization unveils a novel mechanism that crosstalk between ubiquitylation and arginine methylation orchestrates KLF4-mediated genomic stability and carcinogenesis. Our data are consistent with a model in which KLF4 methylation by PRMT5 at a carboxyterminal region, close to the binding site we found for PRMT5, alters the affinity of KLF4 for the E3 ligase VHL. The resulting reduction in KLF4 ubiquitination leads to a stabilization of KLF4 and an increase in cellular KLF4 levels. This in turns leads to enhanced transcription of KLF4 downstream genes, such as *p21* and *Bax*. Since the levels of p21 and Bax are crucial in determining whether cells undergo cell-cycle arrest or apoptosis, the interplay between KLF4 and PRMT5 has important repercussions for tumorigenesis and the DNA damage response (Fig. 8).

Both KLF4 and PRMT5 were proposed to play a role in genome stability. Mouse fibroblasts with targeted deletion of KLF4 are severely sensitive to ionizing radiation and other genotoxic agents because of aberrant DNA damage checkpoint function^39^,^41^,^53^. Furthermore, loss of KLF4 in mouse fibroblasts leads to chromosomal instability even in the absence of exogenous DNA-damaging agents, resulting in aneuploidy as well as other aberrant chromosome abnormalities such as dicentric chromosome, chromatid breaks and double-minute chromosomes^54^.

The physiological relevance of PRMT5 was initially linked to development and cellular proliferation in mouse transgenic study, where targeted deletion of *Prmt5* resulted in early embryonic lethality and suppression of pluripotency in ES cells by rebalancing a set of genes that orchestrate stem cell self-renewal and differentiation^19^. Subsequently, inactivation of *Prmt5* in worm was found to lead to genome instability in response to ionizing radiation^20^,^21^. At the same time, accumulation of PRMT5 in blood, breast, colon and stomach cancers seems to promote cell survival in the face of DNA-damaging agents^23^. Moreover, several critical proteins in oncogentic and apoptotic pathways such as CUL4A/B, EGFR and E2F have been demonstrated to be regulated by PRMT5-mediated methylation^24^,^25^,^26^. During S phase progression, PRMT5 facilitates the mutant cyclin D1/CDK4-mediated suppression of CUL4A/B and thereby stabilizes CDT1, resulting in enhanced DNA replication and cell transformation^24^. In the EGFR signalling pathway, in coordination with EGF-induced EGFR phosphorylation, methylation of EGFR by PRMT5 determines EGF-mediated ERK activation and cell proliferation^25^. In addition, interplay between PRMT5 and PRMT1 on E2F methylation regulates cell growth by rendering the feature of E2F either to promote cell cycle progression or regulate apoptosis^26^. Together, these findings indicate that both KLF4 and PRMT5 regulate genes controlling checkpoints in cell cycle and apoptosis and that aberrant levels of them could lead to failures in genome surveillance. Our findings for the first time report a link between these two genes and provide important insights into their coordination in the maintenance of genome integrity.

Originally identified as a tumour suppressor in several cancers, including in gastrointestinal, oesophageal, lung and pancreatic cancer^7^, KLF4 was found to function as a mitogenic factor in breast cancer and squamous cell carcinoma^8^,^9^,^10^,^11^,^12^,^13^. The initial function of KLF4 as a tumour suppressor (for example, gastric tissue-specific deletion of KLF4 in mice triggers gastric hyperplasia and polyps^55^) was attributed to its transcriptional activation of cyclin-dependent kinase inhibitors such as p21, p27 and p57 (ref. 7). Surprisingly therefore, accumulation of KLF4 has been detected in up to 70% of primary mammary cancers and KLF4 overexpression at the stage of ductal carcinomas suggests that this represents an early event in breast cancer progression carcinoma^8^,^9^,^10^,^11^,^12^. Nuclear localization of KLF4 was found to be a prognostic factor correlating to aggressive phenotype in breast cancers, and knockdown of KLF4 in breast cancer cells induces p53-dependent cell apoptosis, which is consistent with an anti-apoptotic function of KLF4 (refs 1, 2). Furthermore, induction of KLF4 in response to oestrogen receptor stimulation suggests a role of KLF4 in facilitating cell growth through mediating oncogenic signalling in mammary gland epithelial or breast cancer cell^42^. Therefore, depending on the tissue specificity and physiological context, KLF4 can play either a repressive or an active role in tumorigenesis. Efforts so far to explore the underlying mechanism have focused on the interplay between p21, Bax and p53 in malignant transformation using RasV12-induced senescence^11^. The observation in this study adds an additional regulatory layer in orchestrating KLF4 function in cellular transformation through the mechanism of protein methylation (Supplementary Fig. 7B–E), Of note, while both p53 and now KLF4 were found to be substrates of PRMT5, the p53 status does not affect PRMT5 binding to KLF4- or PRMT5-mediated KLF4 stabilization (Supplementary Fig. 8).

Since *Prmt5* knockout in mice results in early embryonic lethality, functional studies for PRMT5 have been limited to cultured-cell model. Elevated PRMT5 expression is detected in a variety of cancers^17^. In breast cancer cells and xenograft breast cancer models, methylation of EGFR by PRMT5 promotes breast cancer cellular proliferation and tumour progression through activation of the ERK-mediated oncogenic pathway^25^.

The stabilization of KLF4 by PRMT5 report here adds a novel explanation to the oncogenic potential of PRMT5 in mammary carcinogenesis. By preventing apoptosis through KLF4-mediated repression of Bax, PRMT5 overexpression inhibits an important checkpoint against tumorigenesis. In scenarios where p21-mediated cell-cycle arrest is not the dominant output of KLF4 elevation, such as when the *CDKN1A* gene is deleted or mutated, PRMT5-mediated stabilization of KLF4 and the resulting alteration in Bax, cyclin D2, cyclin B1, Myc, EGF and IGF transcription will foster tumorigenesis. Furthermore, both KFL4 and PRMT5 play a role in stem cell self-renewal^7^,^19^,^56^, and alteration of both KLF4 or PRMT5 are critical for Slug or Snail-mediated epithelial–mesenchymal transition (EMT)^51^,^52^. It is therefore plausible that alterations in PRMT5 and KLF4 also play a role in metastasis.

Crosstalk between protein modifications has been well characterized, especially in regards to histones and p53 (ref. 57). Presently, we do not know whether there is true crosstalk between KLF4 arginine methylation and ubiquitination, that is, whether ubiquitination of KLF4 also interferes with KLF4 modification by PRMT5. Future studies will also address structural changes caused by KLF4 methylation that inhibit VHL binding. The simulations presented here focused on the mechanism underlying KLF4 recognition by PRMT5 and the catalysis of KLF4 methylation. Acidic PRMT5 residues E483 and E489 seem critical for PRMT5 binding to the KLF4 carboxyterminal docking site containing the basic residues RKR (381–383) near the substrate arginines 374, 376 and 377. Our Molecular Dynamic analysis indicates that PRMT5 catalyses KLF4 methylation as homodimer, with one PRMT5-anchoring KLF4 and the second PRMT5 molecule orienting Zf1 of KLF4 for proper methylation. These detailed insights can provide the basis for rational drug design, hopefully leading to compounds that can be used to alter KLF4 activity—an exciting possibility, given the role of KLF4 in tumorigenesis and the DNA damage response.

## Methods

### Cell lines and cell culture

The HEK293T and HeLaS3 cells were obtained from the American Type Culture Collection. The BTR cells were provided by Dr Daniel S. Peeper (Netherlands Cancer Institute). The p53+/+ and p53−/− HCT116 cells were provided by Lin Zhang. The U2OS cells were provided by Robert W. Sobol. The MCF10A and MCF7 cells were provided by Dr Yi Huang. The viral packaging line Pheonix-A was provided by Dr E.V. Prochownik. The HEK293T, HeLaS3, U2OS, MDA-MB-231 and Pheonix-A cells were maintained in DMEM (Invitrogen) with 10% FBS. The BTR cells were maintained as described previously^11^. The HCT116 cells were maintained in McCoy's 5A (Invitrogen) with 10% FBS.

### Purification of KLF4 complex

HeLa S cells stably expression FLAG-tagged KLF4 were washed twice with PBS and then incubated at room temperature for 30 min with freshly resuspended 2.5 mM DSP (Lomant's reagent). After the crosslinking reaction was stopped by adding Tris pH7.5 to a final concentration of 50 mM for 10 min, nuclear extract from HeLa cells was prepared. KLF4-interacting proteins were purified from the nuclear extract by immunopurification on M2 agarose (Sigma) and washing twice with BC buffer (10% glycerol, 20 mM Tris-HCl [pH 7.9], 0.2 mM EDTA, 0.5 mM phenylmethylsulfonyl fluoride [PMSF], 1 mM DTT, 0.05% NP40) containing 300 mM KCl and once with BC buffer containing 100 mM KCl. The complex was eluted with FLAG peptide in BC buffer containing 100 mM KCl and the resulting elute was treated with 50 mM DTT at 37 °C for 30 min to reverse the crosslinking. The decrosslinked elute was then separated on SDS-PAGE followed by silver staining. The interest bands were cut out for mass spectrum analysis.

### Plasmids and transfection

The lentivirus-based tet inducible system containing pLenti TetR and pLenti CMV/TO Puro DEST #2 was ordered form Addgene. The p21-Luc reporter plasmid was the gift from C. Seiser (Medical University of Vienna). The Lefty1-Luc reporter plasmid was the gift from H. Niwa (Kobe University). The KLF4 full-length and deletion mutant constructs were generated by PCR amplification of the full-length or partial coding sequence of human KLF4 and subsequent subcloning into mammalian expression vectors with FLAG or HA tag. The KLF4 methylation deficient mutant construct was generated using the site-directed mutagenesis kit (Stratagene). The primer sequences are as following, 5′-GGAGCCCAAGCCAAAGAAGGGAAAAAAATCGTGGCCCCGGAAAAG-3′ (forward primer), and 5′-CTTTTCCGGGGCCACGATTTTTTTCCCTTCTTTGGCTTGGGCTCC-3′ (reverse primer). The Myc-tagged PRMT5 full-length and deletion mutant constructs were generated as described previously^57^. The primer sequences for the PRMT5 siRNA resistant mutant construct are as following, 5′-GGACCGTGACCCTGAGGCACAATTCGAAATGCCC TACGTGGTACGGCTGCACAAC-3′ (forward primer), and 5′ GTTGTGCAGCCGTACCACGTA GGGCATTTCGAATTGTGCCTCAGGGTCACGGTCC-3′ (reverse primer). To construct pLenti 6-V5-KLF4 and pLenti CMV/TO-KLF4, the coding sequence of KLF4 was amplified and cloned into pENTR/D-TOPO. The resulting plasmid, pENTR-KLF4 was then used for LR recombination reaction together with the destination vector (ref. 58). For transfection, cells were plated to form a 50–70% confluent culture and then transfected using Lipofectamine 2000 (Invitrogen).

### Lentiviral infection

pLKO.1-PRMT5-shRNA (RHS3979-201825392 from Open Biosystem) or pLenti 6-V5-KLF4 was co-transfected with pVSV-G, pRRE and pRSV-REV into HEK293T cells using Lipofectamine 2000 (ref. 58). The packaged lentiviral particles were collected, mixed with polyprene and added into target cells. The stable cell lines were generated by culturing the cells in the medium containing antibiotic puromycin (1 μg ml^−1^) or blasticidin (10 μg ml^−1^). The KLF4 inducible cell lines (KLF4-WT and KLF4-3K in both MCF10A and MCF7) were generated by sequential infection with pLenti TetR and pLenti CMV/TO-KLF4 and subsequent selection by both blastidicin and puromycin.

### RNA inference

siRNAs (Sigma) specifically targeted to PRMT5 or luciferase were synthesized and transfected into cells using Lipofectamine 2000. Cells were collected at 48 h post-transfection for immunoblotting assay. The synthesized siRNA sequences are as following: Luciferase, 5′- CGT ACGCGGAATACTTCGA-3′; PRMT5, 5′-GCCCAGTTTGAGATGCCTTAT-3′.

### Immunoblotting and immuoprecipitation assay

Cells were harvested and lyzed in RIPA lysis buffer (Upstate Biotechnology) containing protease inhibitor cocktail (Sigma). The protein concentration was determined using Bio-Rad Protein Assay Reagent (Bio-Rad). Immunoblotting was performed using the antibody to KLF4 (sc-20691, 1:1,000), p53 (sc-126, 1:1,000), HA tag (sc-7392, 1:1,000) (Santa Cruz), Myc tag (#2276, 1:1,000), PARP (#9532, 1:1,000), cleaved caspase-3 (#9661, 1:1,000) (Cell Signaling), FLAG tag (F1804, 1:2,000), β-actin (A5441, 1:5,000) (Sigma), PRMT5 (Millipore, 07–405, 1:2,000), p21 (BD Bioscience, 556430, 1:1,000), V5 tag (Invitrogen, R960-25, 1:5,000), and HRP-conjugated goat anti-mouse or anti-rabbit secondary antibody (Promega, W4021 and W4011, 1:5,000). Signals were detected with ECL reagents (Amersham Pharmacia). Semi-quantification of data was performed using software NIH Image J. For immunoprecipitation assay, cells were lyzed in TNE buffer (50 mM Tris-HCl, pH 7.4, 150 mM NaCl, 1 mM EDTA, 0.1% Triton X-100) supplemented with protease inhibitor cocktail. Cell lysates were incubated with the antibody overnight at 4 °C on a rotator. Protein A/G plus agarose (Pierce) was then added and the reactions were further incubated at 4 °C for 2 h. After five washes with TNE buffer without Triton X-100 but supplemented with protease inhibitor cocktail, complexes were released from agarose by boiling for 5 min in 2 × SDS-PAGE loading buffer. Immunoblotting was performed to detect protein expression or ubiquitin conjugates.

### Immunofluorescence

Cells grown on glass bottom culture dishes (MatTek) were transfected with pEGFP-KLF4. Twenty-four hours after transfection, cells were fixed with 4% paraformaldehyde in PBS for 10 min and rendered permeable by further treatment with 0.2% Triton X-100 for 5 min. The PRMT5 antibody (Millipore, 07–405, 1:200) was diluted in PBSB (1% BSA in PBS) and incubated with cells for 1 h. After three washes with PBSB, cells were incubated with Alexa 594-labelled anti-rabbit IgG (Jackson Lab, 111-585-144, 1:500) diluted in PBSB for 1 h. Cells were washed, mounted with UltraCruz DAPI containing mounting medium (Santa Cruz), viewed, and photographed under a FluoView 1200 confocal microscope (Olympus).

### Reporter assay

Cells were plated in 24-well plates. After 24 h, cells were co-transfected with various plasmids together with reporter plasmid and *Renilla* luciferase control vector using Lipofectamine 2000 according to the manufacturer's instruction. Firefly and *Renilla* luciferase activities were measured using a dual luciferase kit (Promega). The firefly luciferase activity for each sample was normalized based on transfection efficiency as determined by *Renilla* luciferase activity. Each experiment was performed in triplicate and repeated at least three times.

### Recombinant KLF4 pull-downs in cell lysates

GST-fused KLF4 wide-type or mutant was expressed in the *E. coli* strain BL21 and purified using glutathione resin following standard protocol. 2 μg of purified recombinant protein was incubated at 4 °C for 1 h with 500 μg of cell lysates from HEK293T cells transfected with HA-PRMT5. Glutathione resin was added and KLF4 interacting protein was pulled down.

### *In vitro* methylation assay

FLAG-tagged PRMT5 was immunoprecipitated from transfected HEK293T cells, incubated with 2 μg of GST-fused KLF4 wide-type or mutant and 2 μCi ^3^H-SAM (Perkin Elmer). Reactions were incubated at 30 °C for 1 h and terminated by addition of SDS sample buffer. Tritiated protein was separated by SDS-PAGE and transferred to polyvinylidene difluoride. For autoradiography, blots were treated with En3Hance Spray (Perkin Elmer) and exposed to film at minus 80 °C for 7 days.

### Annexin V/propidium iodide (PI) assay

Apoptotic cell death was measured by flow cytometric analysis after staining using FITC-conjugated Annexin V/PI Kit (BD PharMingen) following the manufacturer's instructions. In the population of Annexin V-positive cells, PI-negative or PI-positive cells were considered to be early apoptotic or late apoptotic.

### Colony formation assay

BTR cells were seeded in a six-well plates and infected with pLenti 6-V5-KLF4 or pLenti 6-V5-KLF4-mut. Forty-eight hours after infection, the culture medium was supplemented with blasticidin at 10 μg ml^−1^ and changed every three days. After two weeks of culture at 39.5 °C, colonies were fixed with glutaraldehyde (6.0% v/v), stained with crystal violet (0.5% w/v) and counted using a digital colony counter pen.

### Quantitative real time PCR PCR arrays and analysis

Total RNA was isolated from proliferating cells of MCF7 or MCF10A using the Trizol (Invitrogen) according to the manufacturer's instructions. RNA was reversed transcribed using High Capacity RNA-to-cDNA Master Mix (Invitrogen). Real-time PCR was performed on a StepOnePlus Real-Time PCR System (ABI) in duplicates with Fast SYBR Green Master Mix (Applied Biosystems). The cycle threshold (CT) defines the number of PCR cycles required for the fluorescent signal to cross the threshold.

For PCR arrays, the breast cancer genes profile quantitative real-time PCR array PAHS-131Z (Qiagen SABiosciences) plus custom 27 PCR primer were used to simultaneously examine the mRNA levels of 111 genes plus 6 housekeeping control genes. Arrays were performed on StepOne Plus real-time polymerase chain reaction system (Applied Biosystems). Data were analyzed via the comparative CT (^ΔΔ^CT) method and generated heatmap by using the online analysis tools provided by Qiagen SABiosciences (http://sabiosciences.com/pcr/arrayanalysis.php). The resulting values were reported as fold change; only genes showing twofold or greater change were considered.

### *In vivo* tumorigenesis assay

In an orthotopic model, 5 × 10^6^ of MCF-7 cells mixed with matrigel (1:1) were injected into the mammary fat pads of 8-week-old female Crl:Nu-Foxn1(nu) nude mice. Two days before the injection, each mouse was implanted with 17β-estradiol (E_2_) pellets (0.72 mg/pellet) (Innovative Research of America, Sarasota, FL). Tumor size was measured weekly. The animal study was approved by the Institutional Animal Care and Use Committee (IACUC) from University of Pittsburgh.

## Additional information

**How to cite this article:** Hu, D. *et al*. Interplay between arginine methylation and ubiquitylation regulates KLF4-mediated genome stability and carcinogenesis. *Nat. Commun*. 6:8419 doi: 10.1038/ncomms9419 (2015).

## Supplementary Material

Supplementary Information Supplementary Figures 1 -18, Supplementary Tables 1-4, Supplementary Discussion, Supplementary Methods and Supplementary References.

Supplementary Movie 1 Protocol for the series of MD simulations performed to generate the PRMT5-KLF4 complex
