# Supplementary material for: Interplay between arginine methylation and ubiquitylation regulates KLF4-mediated genome stability and carcinogenesis
Source: Nat Commun. 2015 Sep 30;6:8419. doi: 10.1038/ncomms9419 (PMC4598737; doi:10.1038/ncomms9419)
Supplement: Supplementary Information — Supplementary Figures 1 -18, Supplementary Tables 1-4, Supplementary Discussion, Supplementary Methods and Supplementary References. [file ncomms9419-s1.pdf]

A

gi|2323410 Mass: 73424 Score: 400 Queries matched: 85 emPAI: 0.48
SkblHs [Homo sapiens]

Query	Observed	Mr(expt)	Mr(calc)	ppm	Miss	Score	Expect	Rank	Peptide
261	653.3560	652.3487	652.3432	8.52	0	41	0.0044	1	R.SYTIGL.- 254 255 256 257 258 259 260 262 263 264 265
1243	961.5050	960.4977	960.4916	6.35	0	26	0.42	1	R.EFIQEPAK.N 1244
1247	482.3014	962.5883	962.5800	8.56	1	24	0.074	1	R.IKLYAVEK.N 1245 1246 1248
1523	1045.5741	1044.5668	1044.5604	6.16	0	14	5	1	R.DWNTLIVGK.L 1471 1482 1485 1488 1495 1498 1515 1516 1525
1864	599.8372	1197.6599	1197.6506	7.81	0	26	0.23	1	K.LSPWIRPDSK.V 1857 1858 1859 1860 1861 1862 1863 1865 1866 1867 1868
2032	646.3233	1290.6321	1290.6244	5.96	0	41	0.0097	1	K.YSQYQQAIYK.C 2031 2033 2034 2035
2180	694.9164	1387.8182	1387.8075	7.69	0	33	0.014	1	K.AAILPTSIFLTNK.K 2181 2182 2183
2463	777.9437	1553.8728	1553.8566	10.5	1	20	0.42	1	K.LSPWIRPDSKVEK.I 2461 2462 2464
2709	893.4628	1784.9110	1784.8978	7.40	0	48	0.0018	1	R.DLNCVPEIADTLGAVAK.Q 2669 2675 2676 2677 2686 2687 2692 2693 2694 26
3049	1085.5440	2169.0734	2169.0477	11.8	0	44	0.0038	1	K.DDGVSIPGEYTSFLAPISSSK.L 3047 3048
3118	1136.5941	2271.1737	2271.1529	9.18	1	45	0.0026	1	R.VSSGRDLNCVPEIADTLGAVAK.Q 3116 3117 3119 3120

Proteins matching the same set of peptides:
gi|20070220 Mass: 73322 Score: 400 Queries matched: 85
protein arginine methyltransferase 5 isoform a [Homo sapiens] **PRMT5**

http://ms-searching2.rockefeller.edu/mascot/cgi/master_results.pl?file=../data/20090204/F0... 2/4/2009

B

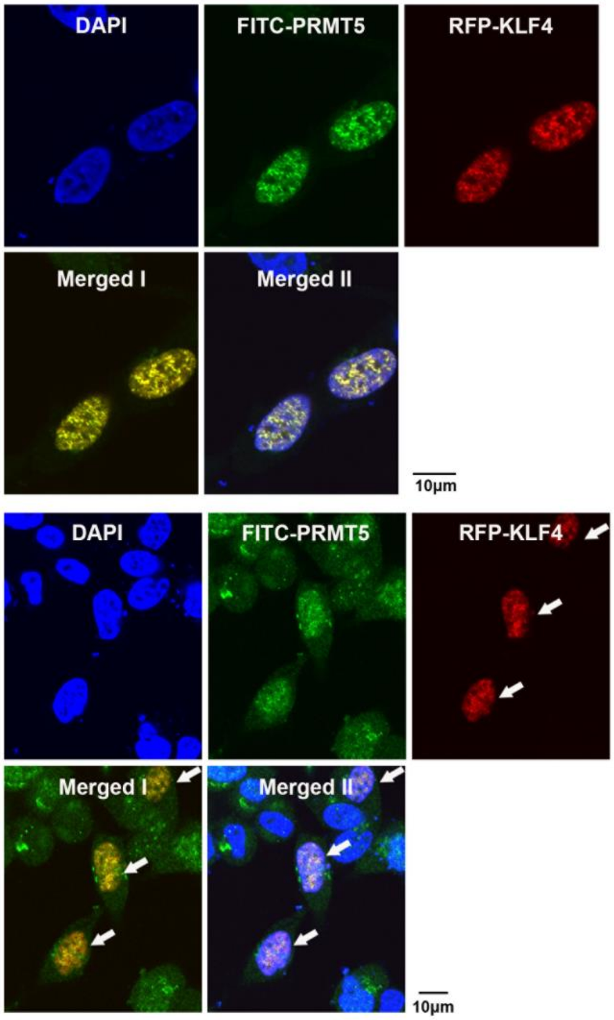

Supplementary Figure 1. Mass spectrometric analysis and validation using immunostaining. (**A**) Result of mass spectrometric analysis indicating the interaction between KLF4 and PRMT5. (**B)** Validation of the interaction between KLF4 and PRMT5 by immunostaining. pEGFP-KLF4 was transfected into HCT116 (upper panel) and HEK293 cells (lower panel). The endogenous PRMT5 was detected by confocal immunofluorescence microscopy using the PRMT5 antibody with the nuclei staining by DAPI. Scale bar, 10 μm.

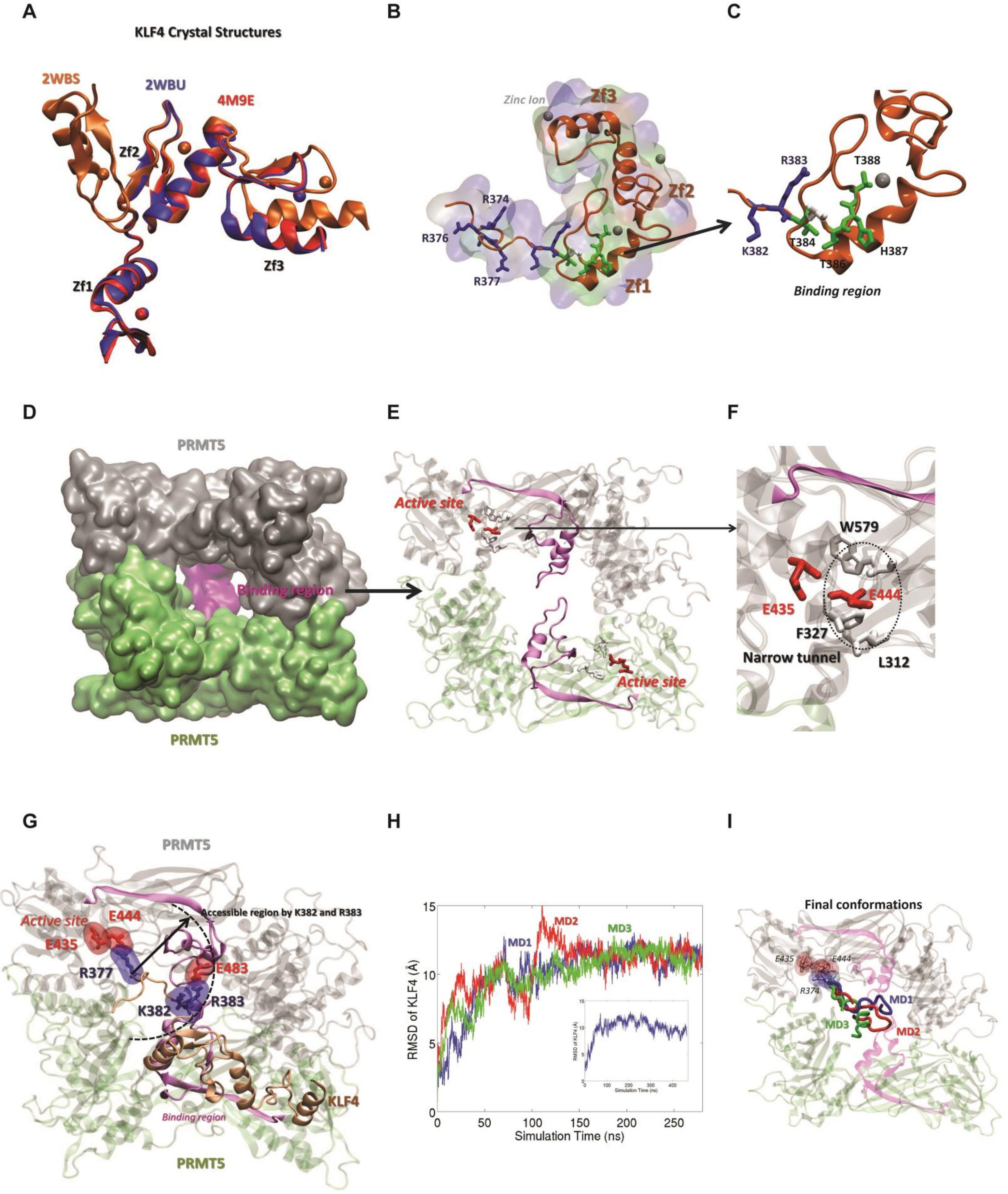
A
KLF4 Crystal Structures
2WBS
2WBU
4M9E
Zf2
Zf1
Zf3
B
Zinc Ion
Zf3
Zf2
Zf1
R374
R376
R377
C
R383
T388
K382
T384
T386
H387
Binding region
D
PRMT5
Binding region
PRMT5
E
Active site
Active site
F
W579
E435
E444
F327
Narrow tunnel
L312
G
PRMT5
Accessible region by K382 and R383
Active site
E444
E435
R377
E483
K382
R383
Binding region
KLF4
PRMT5
H
MD1
MD2
MD3
RMSD of KLF4 (Å)
Simulation Time (ns)
I
Final conformations
E435
E444
R374
MD1
MD3
MD2

Supplementary Figure 2. Structures of KLF4 and PRMT5. (A) KLF4 crystal structures 2WBS (orange), 2WBU (blue) and 4M9E (red) superimposed with respect to their second zinc fingers (Zf2). **(B)** KLF4 conformer after 80ns of MD simulation. Residues R374, R376 and R377, which our experiments showed to be methylated, and the experimentally predicted binding region, K382-T388, are shown in licorice representation. **(C)** First zinc finger in panel **(B)** is enlarged. The transparent-surface and the licorice representations are colored according to their residue types; non-polar (white), basic (blue), acidic (red) and polar (green). **(D-F)** Human PRMT5 homodimer conformer after 30ns of MD simulation. **(D)** PRMT5 dimer in surface representation. Experimentally predicted binding region (magenta) is located at the bottom of the large crevice formed by the two PRMT5s. **(E)** shows the same conformer in cartoon representation, where PRMT5s except their binding regions are shown in transparent. **(F)** Key residues at the active site. E435 and E444, and the residues constructing the narrow entrance tunnel to the active site, L312, F327 and W579, are shown in licorice representation and colored according to their residue types. The narrow entrance tunnel is shown in a cartoonish manner with a circle. **(G)** Initial bound conformation based on the surface chemical properties with R377 being in the active site. R377 is in the active site of PRMT5 interacting with E435 and E444. K382 and R383 of KLF4 are being in a close proximity of the oppositely charged E483 of PRMT5. A cartoonish representation of the accessible region on PRMT5 to the KLF4 residues K382 and R383 is shown with a dashed arc. **(H-I)** Simulation of the partial KLF4 structures (residues P370-T388) bound to PRMT5 dimer. 3 runs were performed; MD1, MD2 and MD3. All conformers were superimposed with respect to their PRMT5 dimers to emphasize the change in the relative position of KLF4 to the PRMT5 dimers. **(H)** The backbone atom RMSDs of KLF4 from their starting positions. **(I)** Binding modes obtained from simulations. Residues E435 (PRMT5), E444 (PRMT5), and R374 (KLF4) are shown in licorice representation to show that interaction in the active site are conserved.

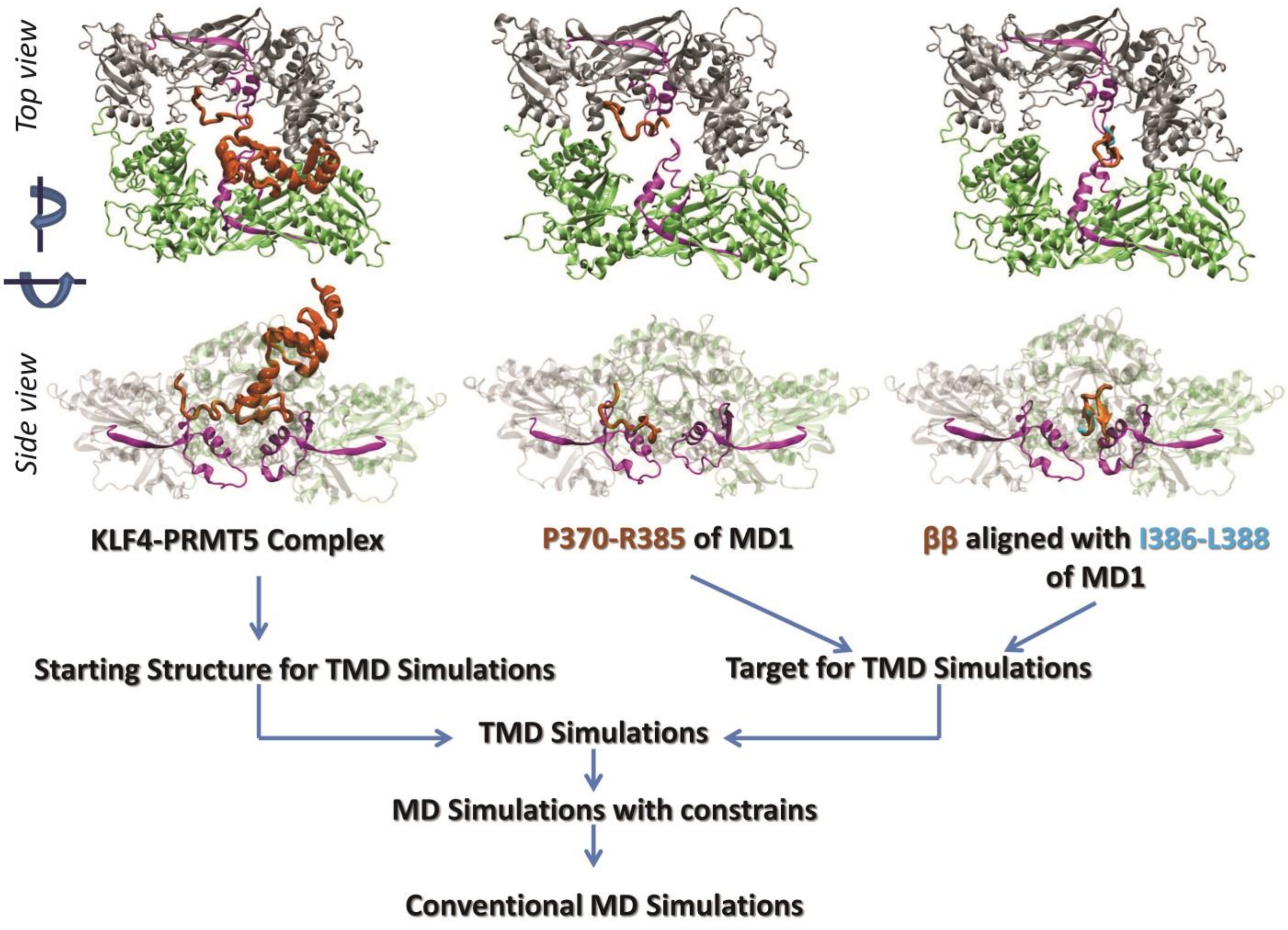

Supplementary Figure 3. Refining Binding Mode based on MD1. The starting conformer for the tMD simulations is shown on the left top corner. The coordinates, taken from the final conformer of MD1, of the backbone residues P370-R385 of KLF4 are shown in the upper-middle panel. The ββ structure of Zf1 in KLF4 (KLF4-PRMT5 complex) was aligned with the backbone atoms of the residues I386-L388 (shown in cyan) of the final conformer of MD1. The C^a coordinates of the aligned KLF4 residues (shown in orange in the upper-right panel) are given as targets for tMD simulations. Details of the simulations are provided in Supplementary Table 2.

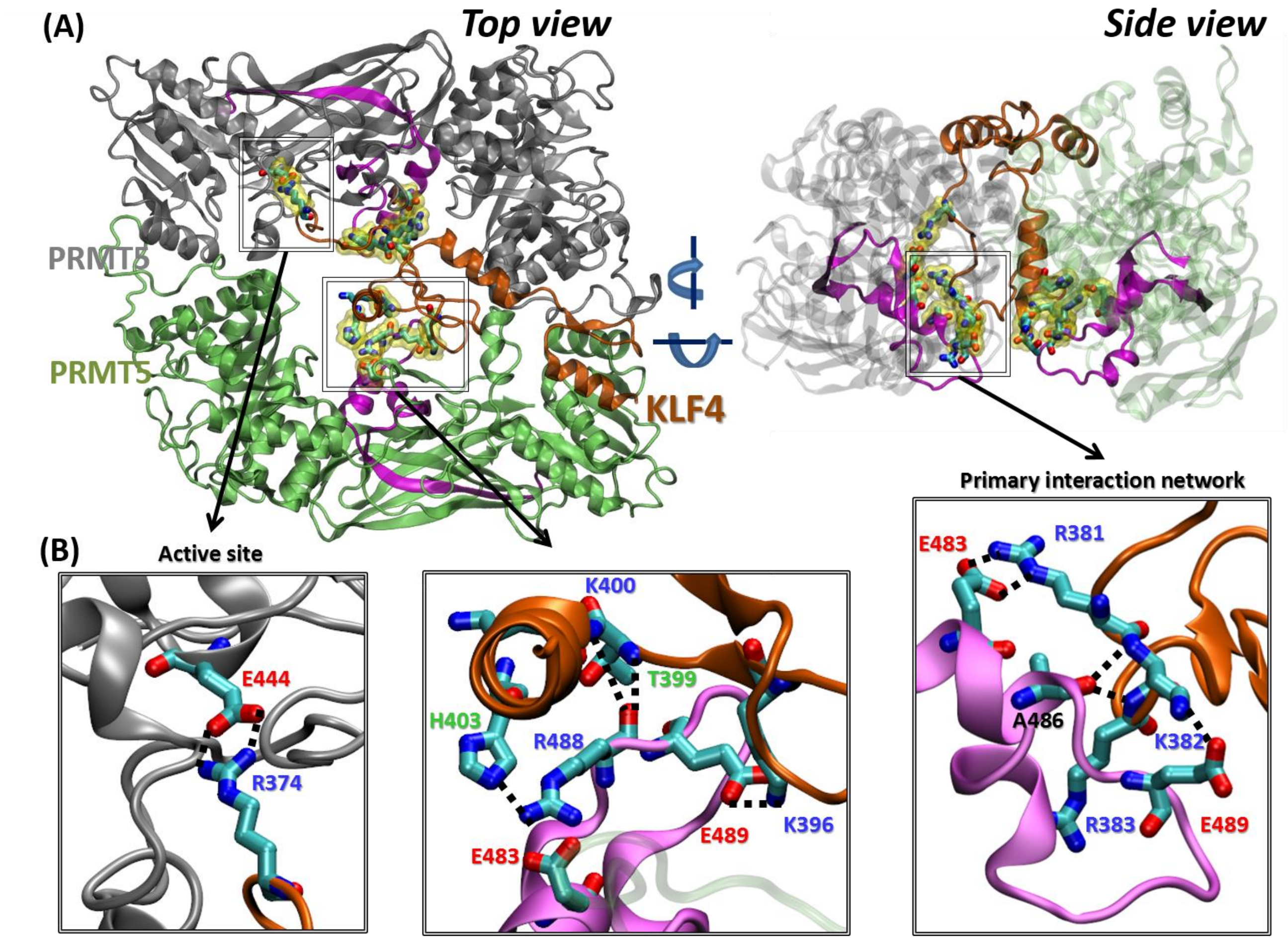

Supplementary Figure 4. PRMT5-KLF4 complex from the second run. (A) The top and side views of the complex obtained from the second run (after 230ns MD simulation) is shown at the top. PRMT5s are shown in grey and lime. The experimentally predicted interaction region on PRMT5 is shown in *magenta*. KLF4 is shown in orange. **(B)** In the lower panel on the left the interaction between KLF4 residue R374 and PRMT5 residue E444 at the active site are shown. In the lower middle panel interactions between Zf1 (excluding the experimentally predicted binding region) and the experimentally predicted PRMT5 binding region are shown. On the lower right panel the primary interaction network, which is closely located to the methylated arginines of KLF4, between the experimentally predicted binding regions are shown. Residues interacting are shown in licorice representation and colored according to their atom types.

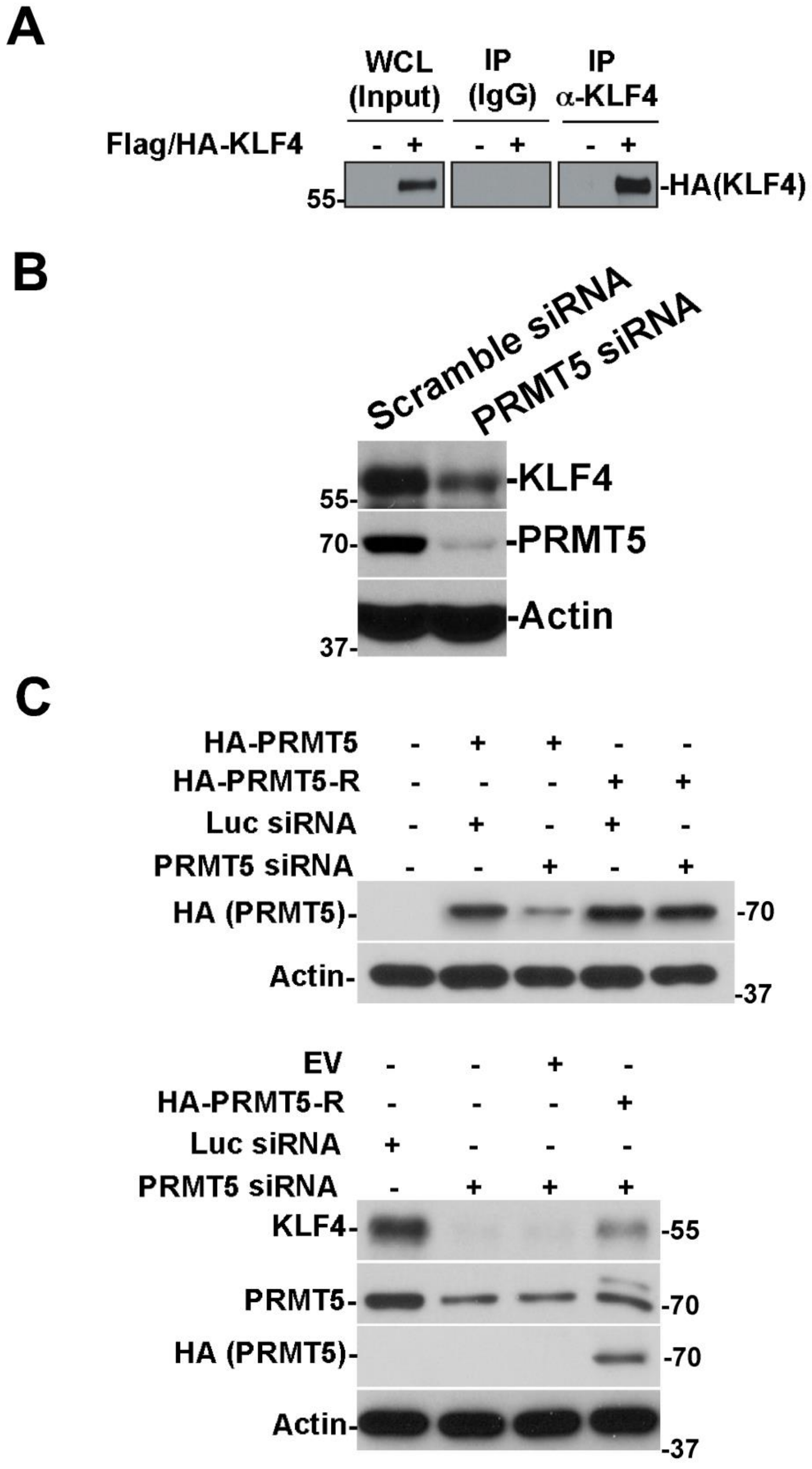
A
WCL (Input)
IP (IgG)
IP α-KLF4
Flag/HA-KLF4
- + - + - +
55-
-HA(KLF4)
B
Scramble siRNA
PRMT5 siRNA
-KLF4
55-
70-
-PRMT5
-Actin
37-
C
HA-PRMT5 - + + - -
HA-PRMT5-R - - - + +
Luc siRNA - + - + -
PRMT5 siRNA - - + - +
HA (PRMT5)-
-70
Actin-
-37
EV - - + -
HA-PRMT5-R - - - +
Luc siRNA + - - -
PRMT5 siRNA - + + +
KLF4-
-55
PRMT5-
-70
HA (PRMT5)-
-70
Actin-
-37

Supplementary Figure 5. KLF4 downregulation is a specific effect caused by PRMT5 siRNA. (**A**) Examination of immunoprecipitation efficiency for polyclone antibody against KLF4. The molecular weight of endogenous KLF4 runs approximately 55 KDa, which completely overlaps with the heavy chain of IgG and masks each other. Thus, we alternatively used this polyclone antibody to pull down ectopic expressed KLF4 (HA tag) and detected the IP efficiency by blotting with anti-HA antibody. (**B**) PRMT5 depletion leads to KLF4 protein downregulation. MCF7 cells were transfected with PRMT5 siRNA or control luciferase siRNA. Cell lysates were immunoblotted with the antibody against PRMT5 or KLF4. β-actin was used as loading control. (**C**) PRMT5 re-expression rescues KLF4 expression in PRMT5-KD cells. Upper panel, validation of PRMT5 siRNA resistant mutant. PRMT5 wide-type or siRNA resistant mutant was co-transfected with PRMT5 siRNA or control luciferase siRNA into HEK293T cells. Cell lysates were immunoblotted with the HA tag antibody. Lower panel, PRMT5 siRNA resistant mutant or empty vector was co-transfected with PRMT5 siRNA or control luciferase siRNA into HCT116 cells. Cell lysates were immunoblotted with the antibody against KLF4, PRMT5, or HA.

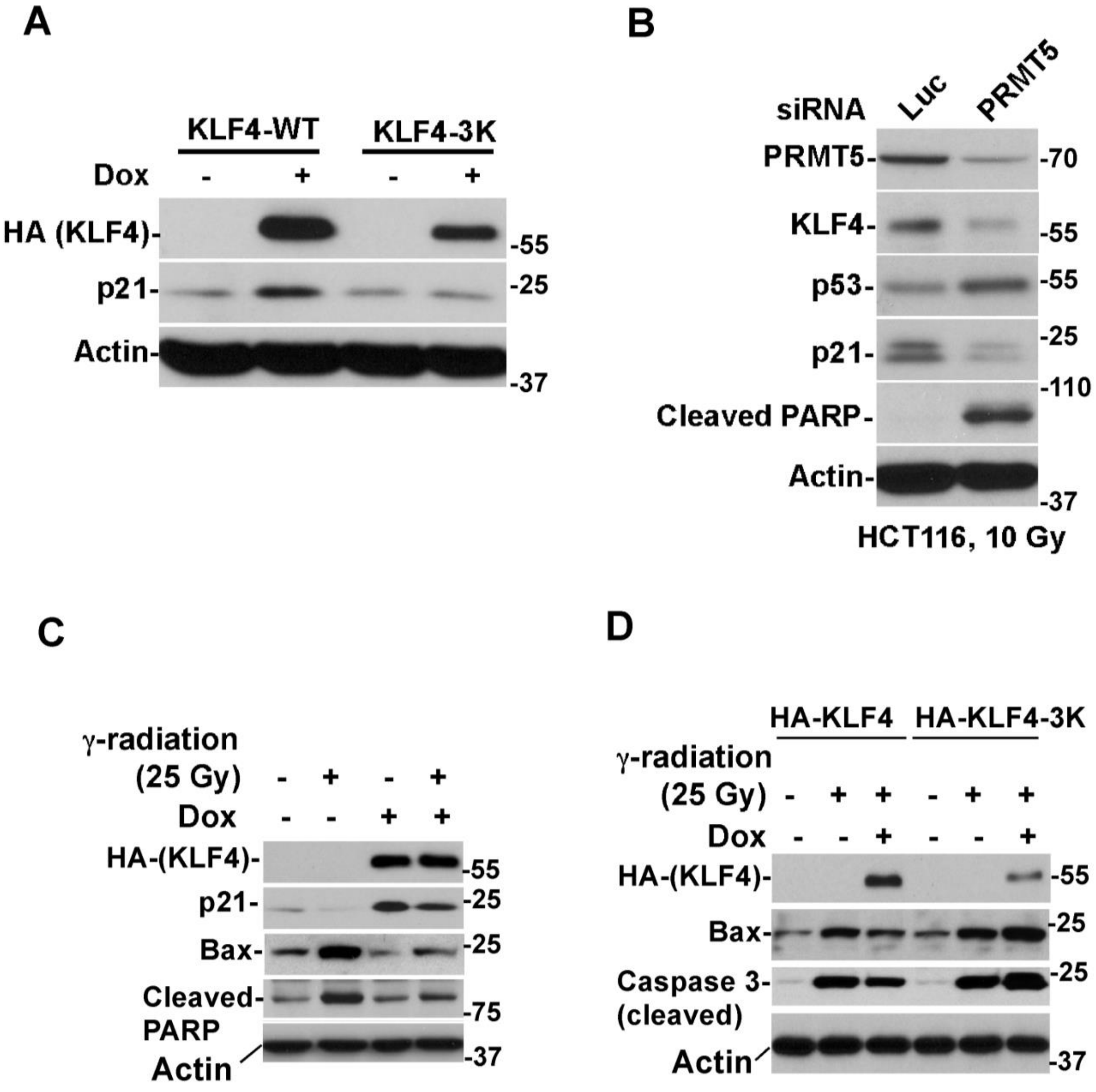
A
KLF4-WT
KLF4-3K
Dox - + - +
HA (KLF4)-
-55
p21-
-25
Actin-
-37
B
siRNA
Luc
PRMT5
PRMT5-
-70
KLF4-
-55
p53-
-55
p21-
-25
-110
Cleaved PARP-
Actin-
-37
HCT116, 10 Gy
C
γ-radiation
(25 Gy) - + - +
Dox - - + +
HA-(KLF4)-
-55
p21-
-25
Bax-
-25
Cleaved-
PARP
-75
Actin
-37
D
HA-KLF4
HA-KLF4-3K
γ-radiation
(25 Gy) - + + - + +
Dox - - + - - +
HA-(KLF4)-
-55
Bax-
-25
Caspase 3-
(cleaved)
-25
Actin
-37

Supplementary Figure 6. PRMT5 depletion leads to KLF4 downregulation followed by apoptosis induction. (**A**) KLF4 methylation deficient mutant loses the ability to induce p21 expression. KLF4 inducible U2OS cells were treated with 10nM doxycycline for 8 hrs and harvested for immunoblotting with the antibody against HA or p21. (**B**) PRMT5 siRNA or control luciferase siRNA was transfected into U2OS cells. 24 hrs after transfection, cells were treated with 10 Gy of γ-radiation for additional 24 hrs and cell lysates were immunoblotted with the antibody agaist PRMT5, KLF4, p21 or cleaved PARP. (**C**) Under lethal dose (25 Gy γ-radiation), elevation of KLF4 results in the suppression of γ-radiation-induced cellular apoptosis. (**D**) Disruption of KLF4 methylation leads to the downregulation of KLF4, which compromised the KLF4-governed suppression of apoptosis. KLF4 inducible U2OS cells were treated with 2 nM of doxycycline for 16 hrs and then γ-radiation (25 Gy) for 16h. Cell lysates were immunoblotted with the antibody to HA and cleaved caspase-3.

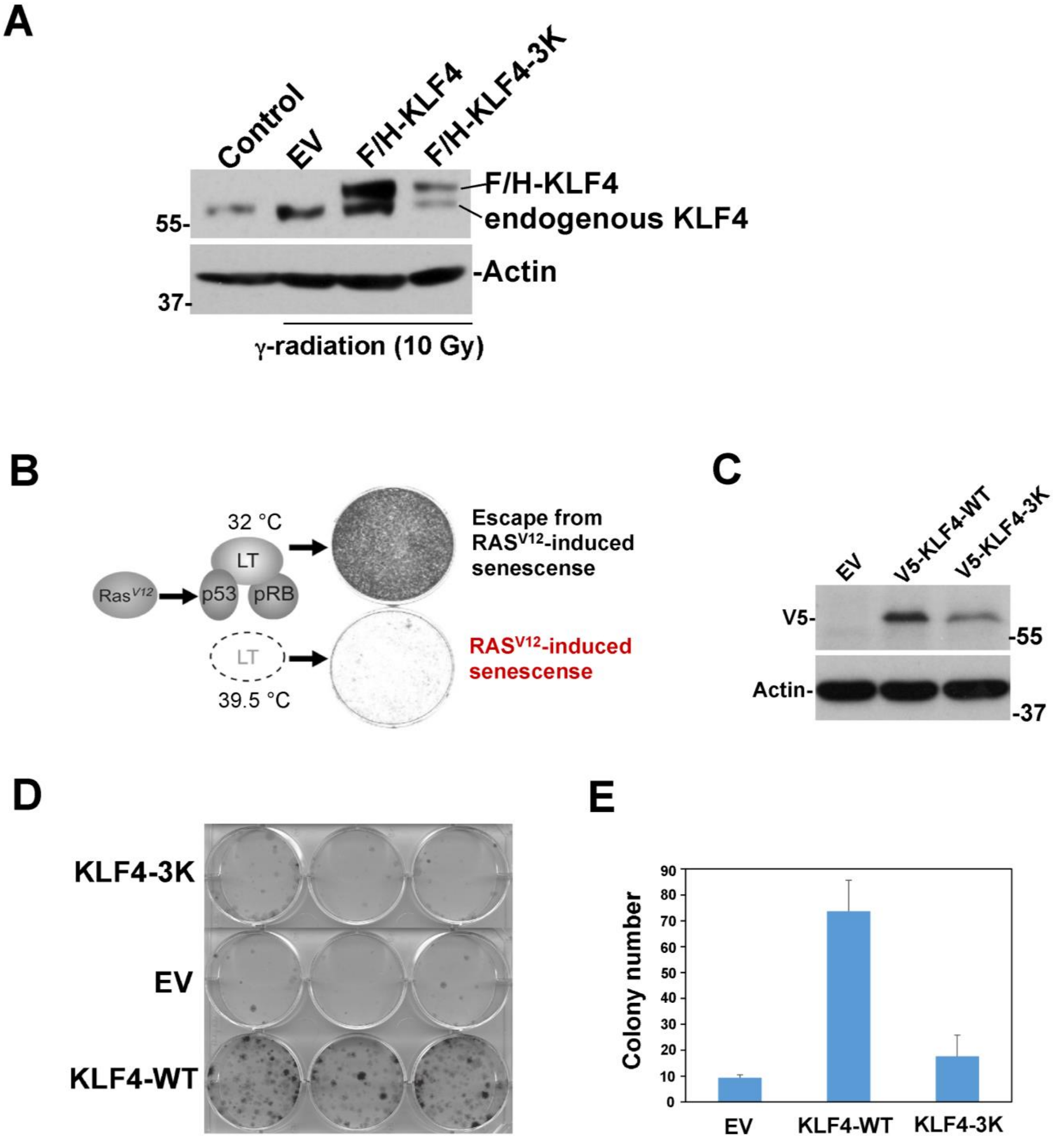
A
Control
EV
F/H-KLF4
F/H-KLF4-3K
F/H-KLF4
endogenous KLF4
55-
-Actin
37-
γ-radiation (10 Gy)
B
32 °C
LT
Ras^{V12}
p53
pRB
Escape from RAS^{V12}-induced senescense
LT
39.5 °C
RAS^{V12}-induced senescense
C
EV
V5-KLF4-WT
V5-KLF4-3K
V5-
-55
Actin-
-37
D
KLF4-3K
EV
KLF4-WT
E
Colony number
0
10
20
30
40
50
60
70
80
90
EV
KLF4-WT
KLF4-3K

Supplementary Figure 7. PRMT5-dependent methylation is critical for KLF4-mediated cellular transformation. (A) Functional interference of KLF4 by methylation resistant mutant KLF4. The same experiments were designed as described in Figure 5I. To measure the expression levels of both endogenous and exogenous KLF4 before or after the treatment with γ-radiation, lysates were prepared from aliquot of same batch of cell collection as described in Figure 5I. Expression levels of endogenous or ectopically expression mutant KLF4 were measured by using antibodies against either KLF4 or Flag. (**B**) Schematic representation of BTR cell system. BTR cells stably expresses Ras^{V12} and a temperature-sensitive mutant of SV40 large T antigen (LT). Cells proliferate rapidly at the permissive temperature (32°C) but undergo premature senescence at the restrictive temperature (39.5°C). (**C**) Ectopic KLF4 expression in BTR cells. BTR cells were infected with lentivirus expressing V5-tagged KLF4 wide-type or mutant and harvested for immuoblotting with the V5 tag antibody. (**D**) Elevated KLF4 bypasses the Ras^{V12} –induced senescence, while failure of KLF4 methylation attenuates KLF4-drived colony formation. BTR cells were infected with lentivirus expressing V5-tagged KLF4 wide-type or mutant and then selected with puromycin for 14 days. The colonies were fixed, stained with crystal violet and counted. (**E**) The number of colony from **C** is presented. Values represent mean ± S.E.M. from triplicate.

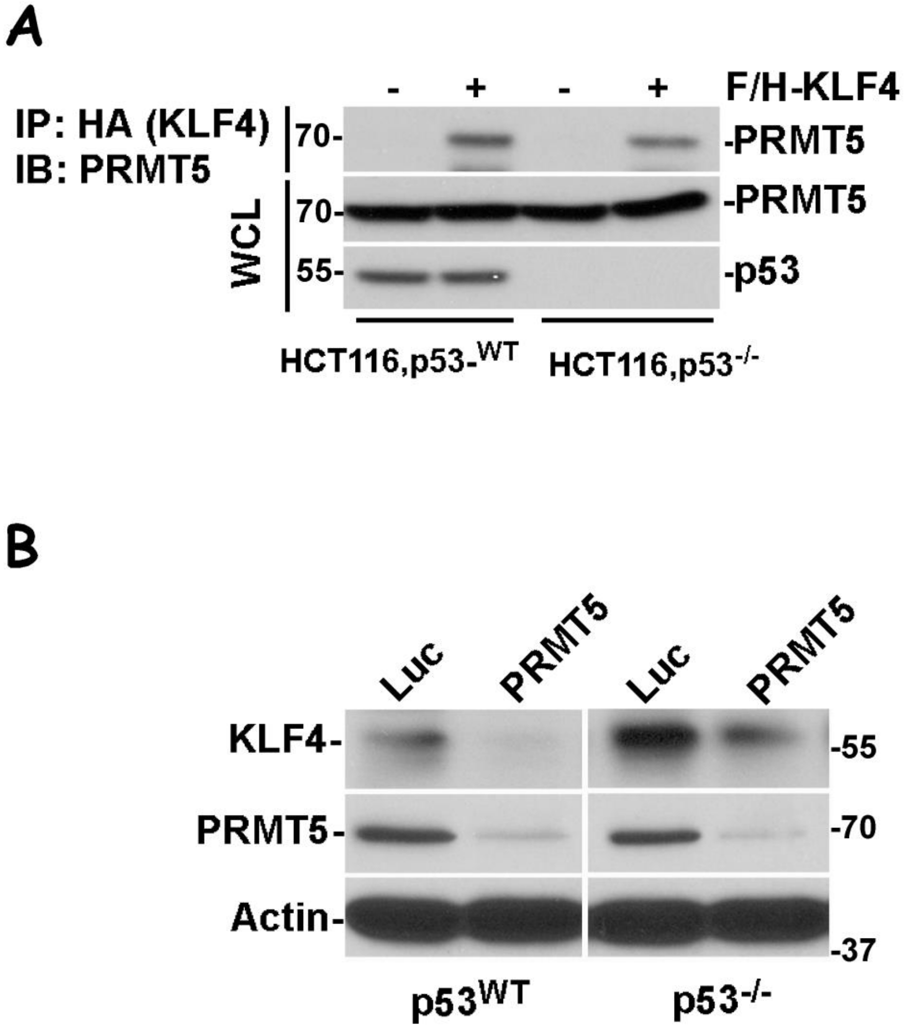

Supplementary Figure 8. Both PRMT5-KLF4 binding and KLF4 regulation by PRMT5 are p53-independent. (**A**) PRMT5 binding to KLF4 is p53-independent. Flag and HA-tagged KLF4 was transfected into both HCT116 *p53*+/+ and HCT116 *p53*-/- cells. Cell lysates were immunoprecipitated with the HA tag antibody and then immunoblotted with the PRMT5 antibody. Levels of input protein are shown. (**B**) P53 depletion doesn't affect the regulation of KLF4 expression by PRMT5. PRMT5 siRNA or control luciferase siRNA was transfected into both HCT116 *p53*+/+ and HCT116 *p53*-/- cells. Cell lysates were immunoblotted with the antibody against KLF4 or PRMT5.

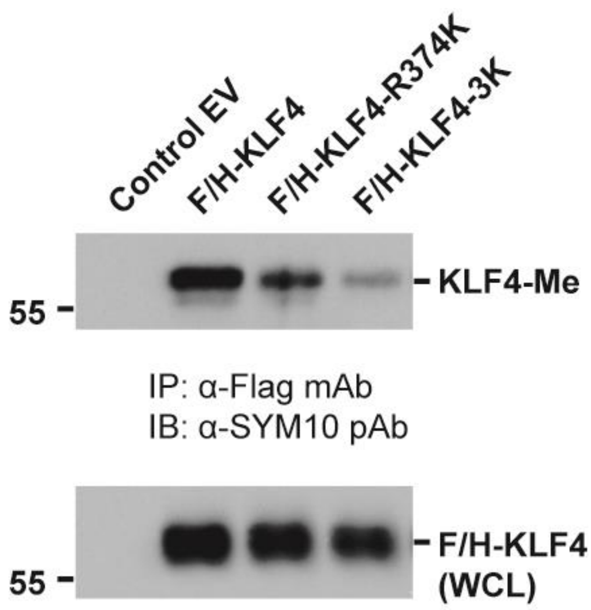

Supplementary Figure 9. Mutation of arginine methylation sites inhibits KLF4 methylation. HEK293T cells were transfected with Flag and HA-tagged KLF4-WT or mutant (R374K or 3K). The cell lysates were immunoprecipitated with the Flag tag antibody and then immunoblotted with the antibody SYM10.

Figure 1

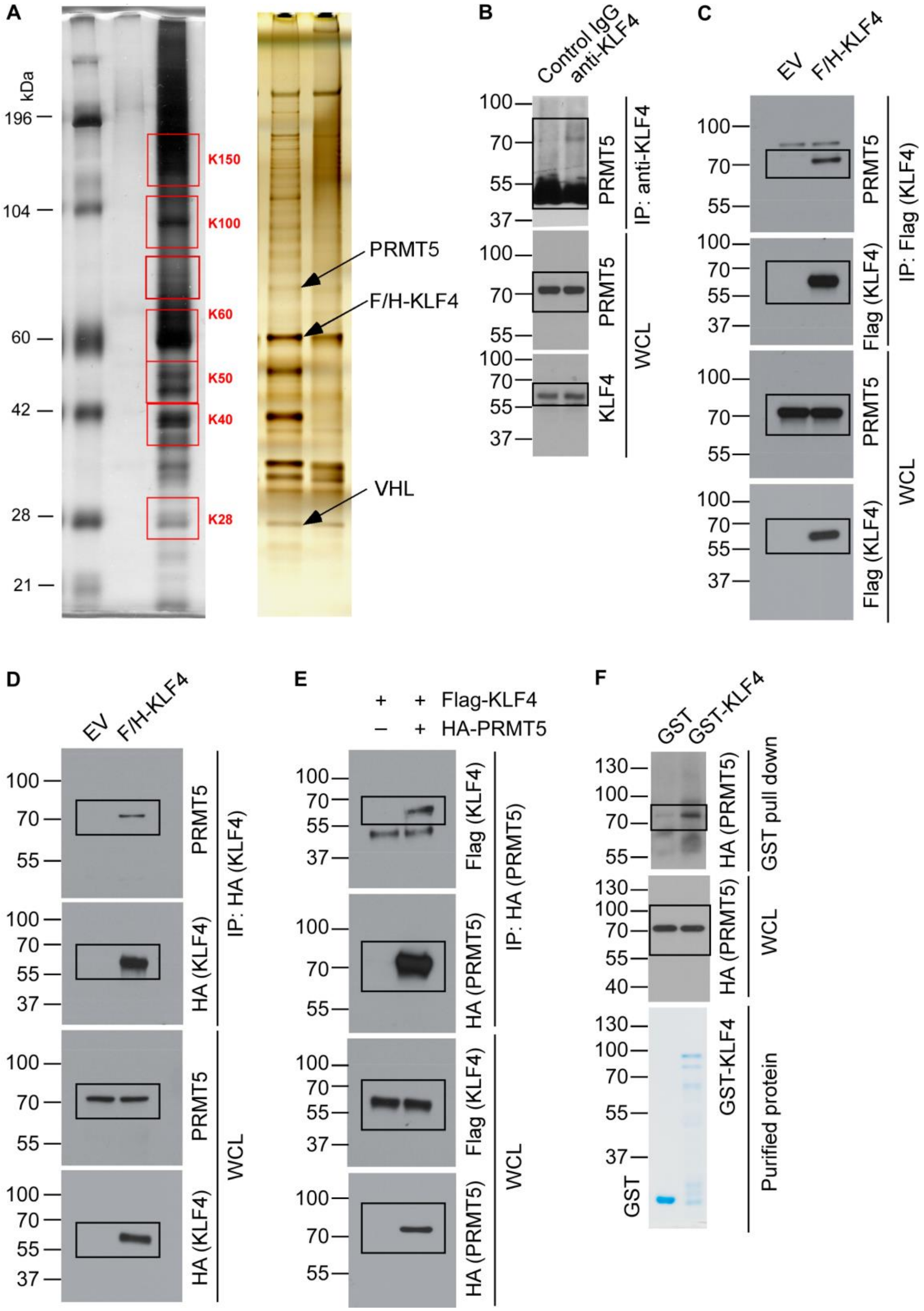

Supplementary Figure 10. Full Scans of Western blots

Figure 2

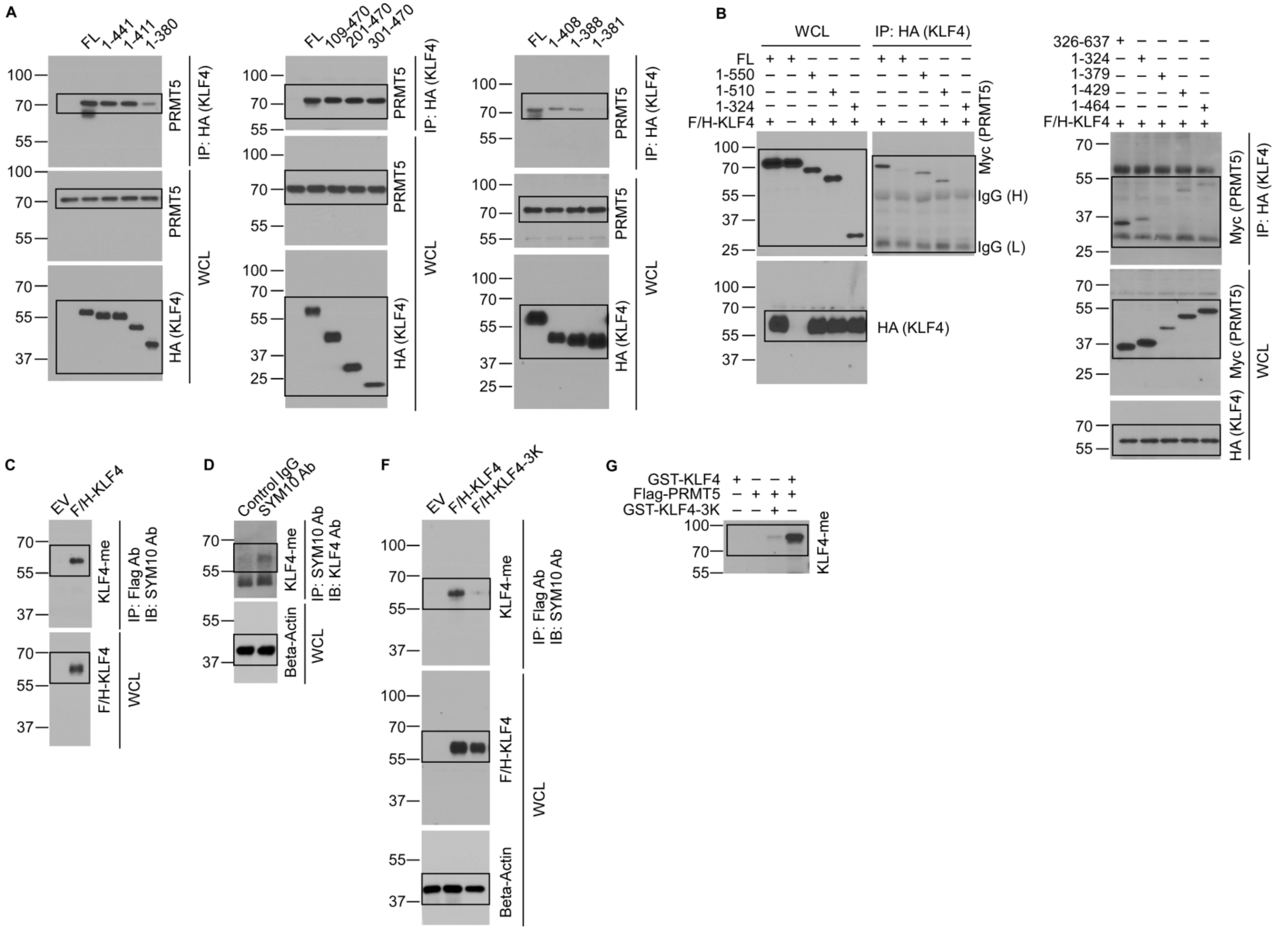

Supplementary Figure 11. Full Scans of Western blots

Figure 4

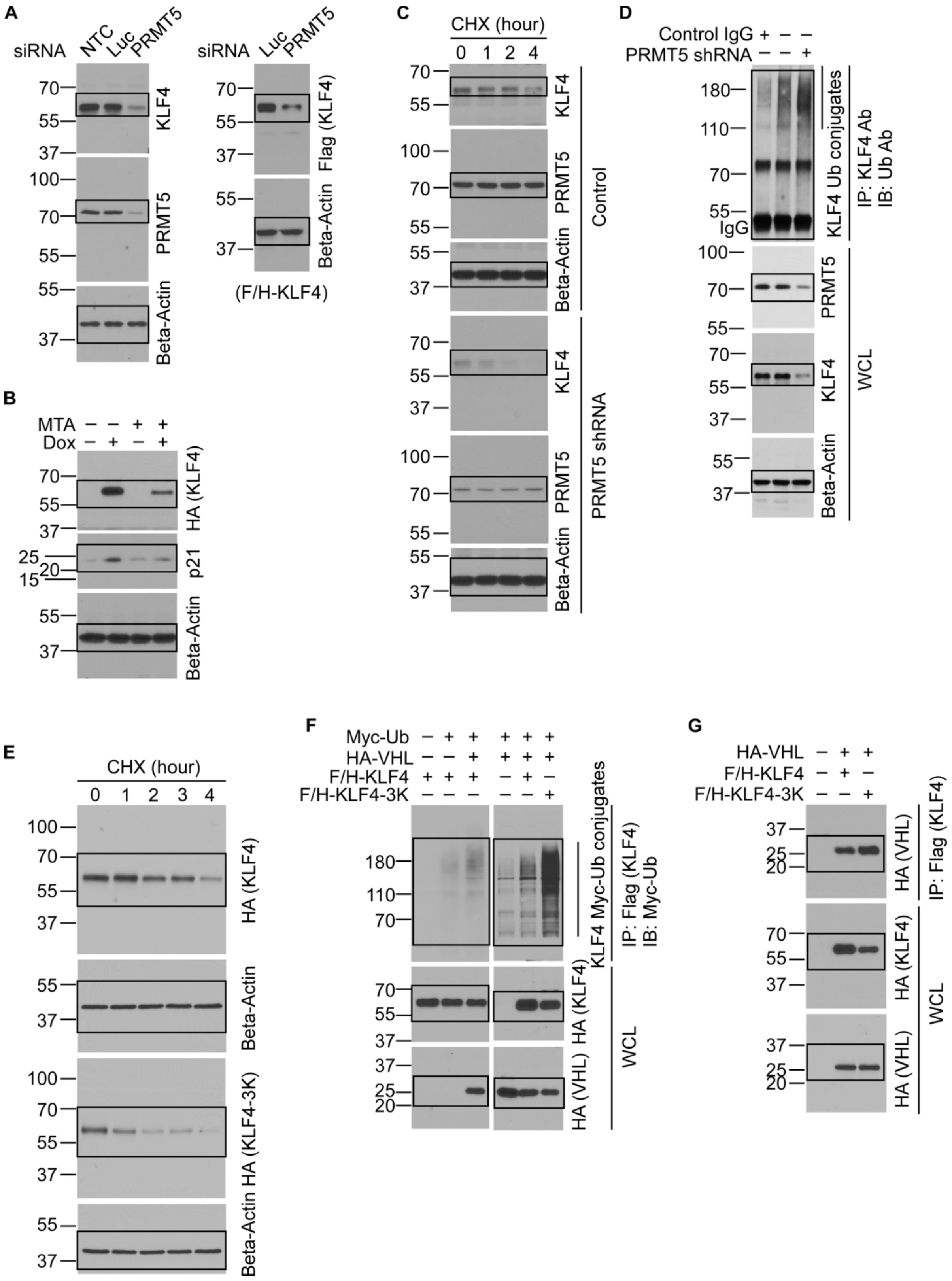

Supplementary Figure 12. Full Scans of Western blots

Figure 5

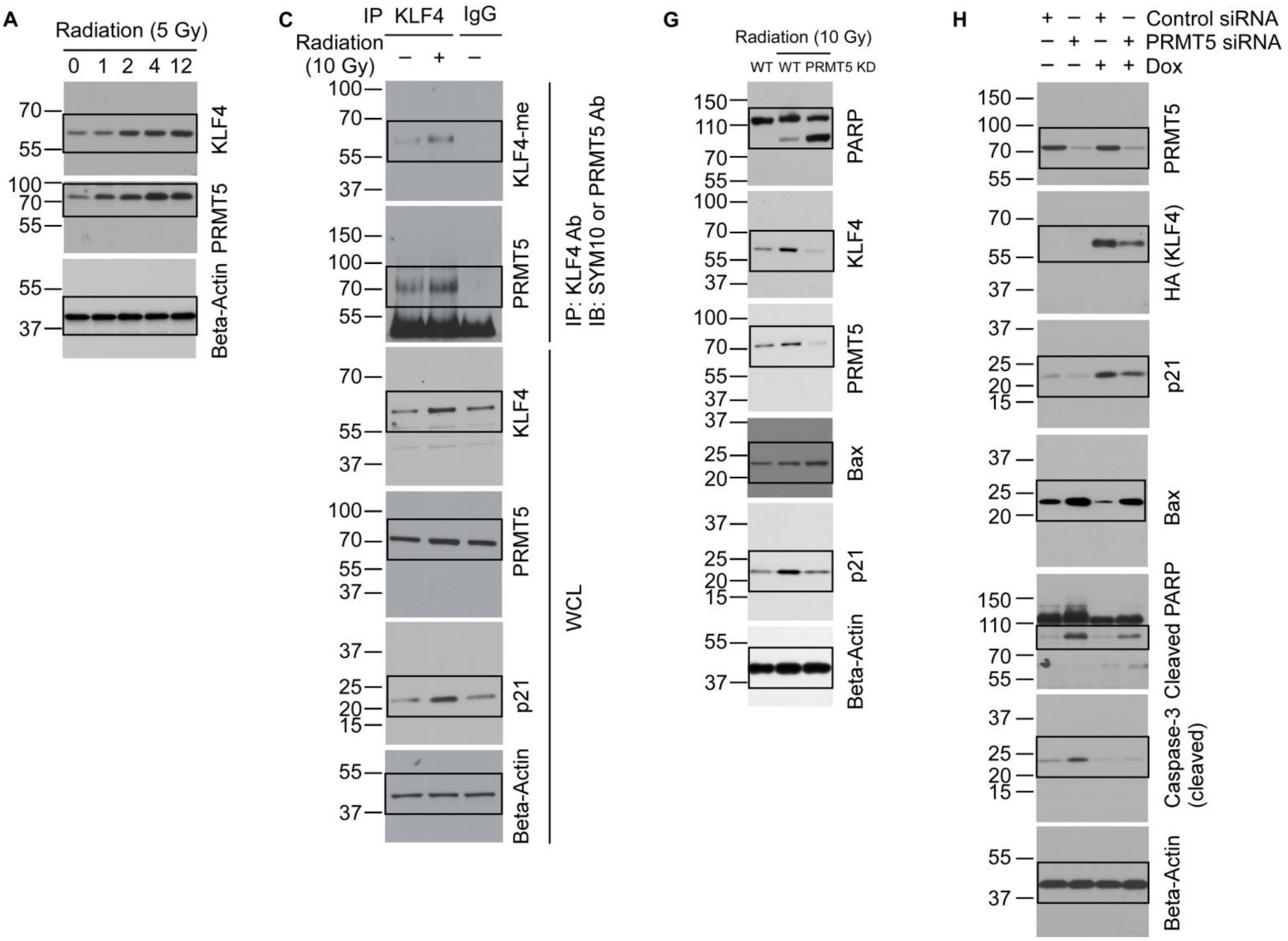

Supplementary Figure 13. Full Scans of Western blots

Supplementary Figure 5

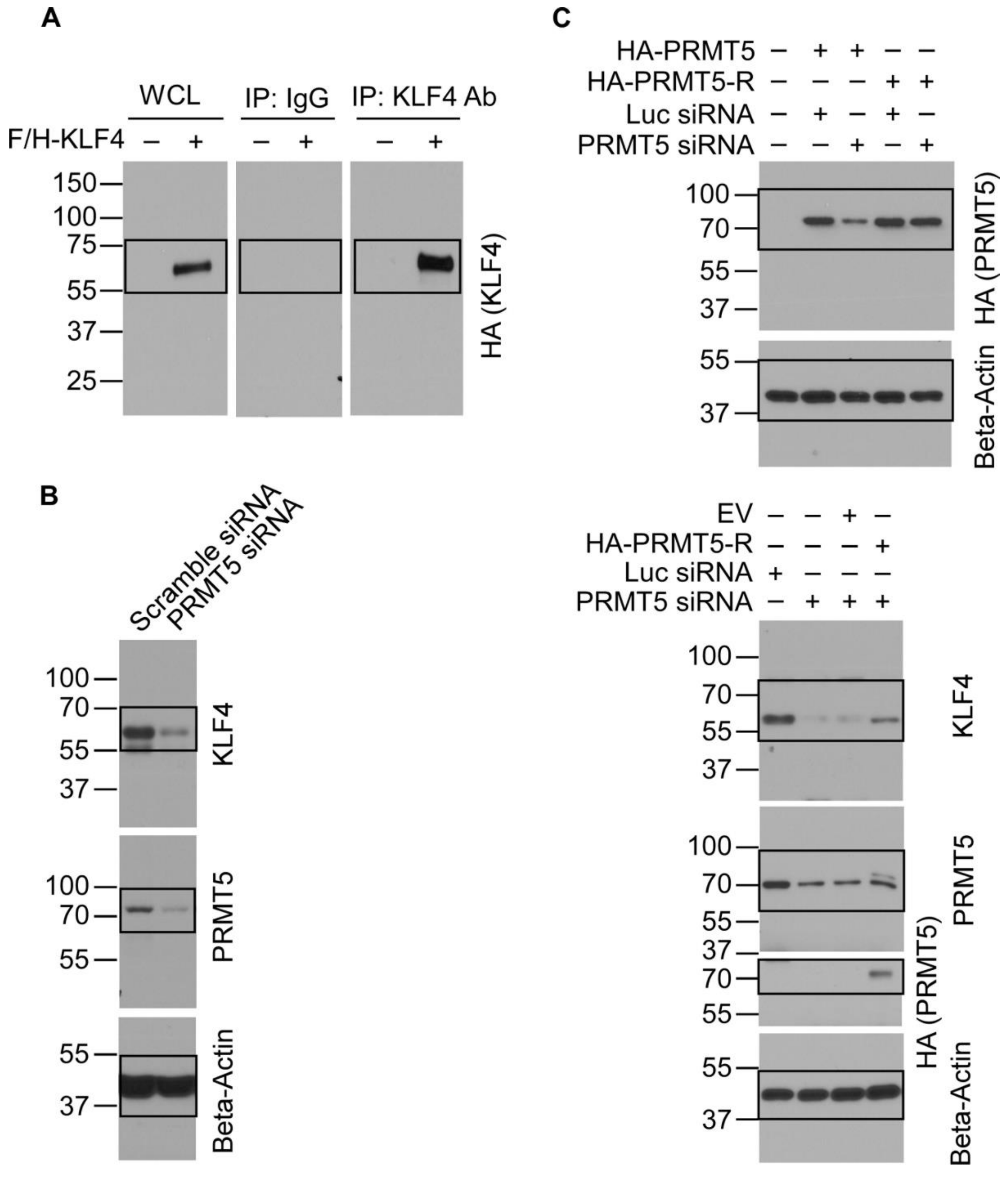

Supplementary Figure 14. Full Scans of Western blots

Supplementary Figure 6

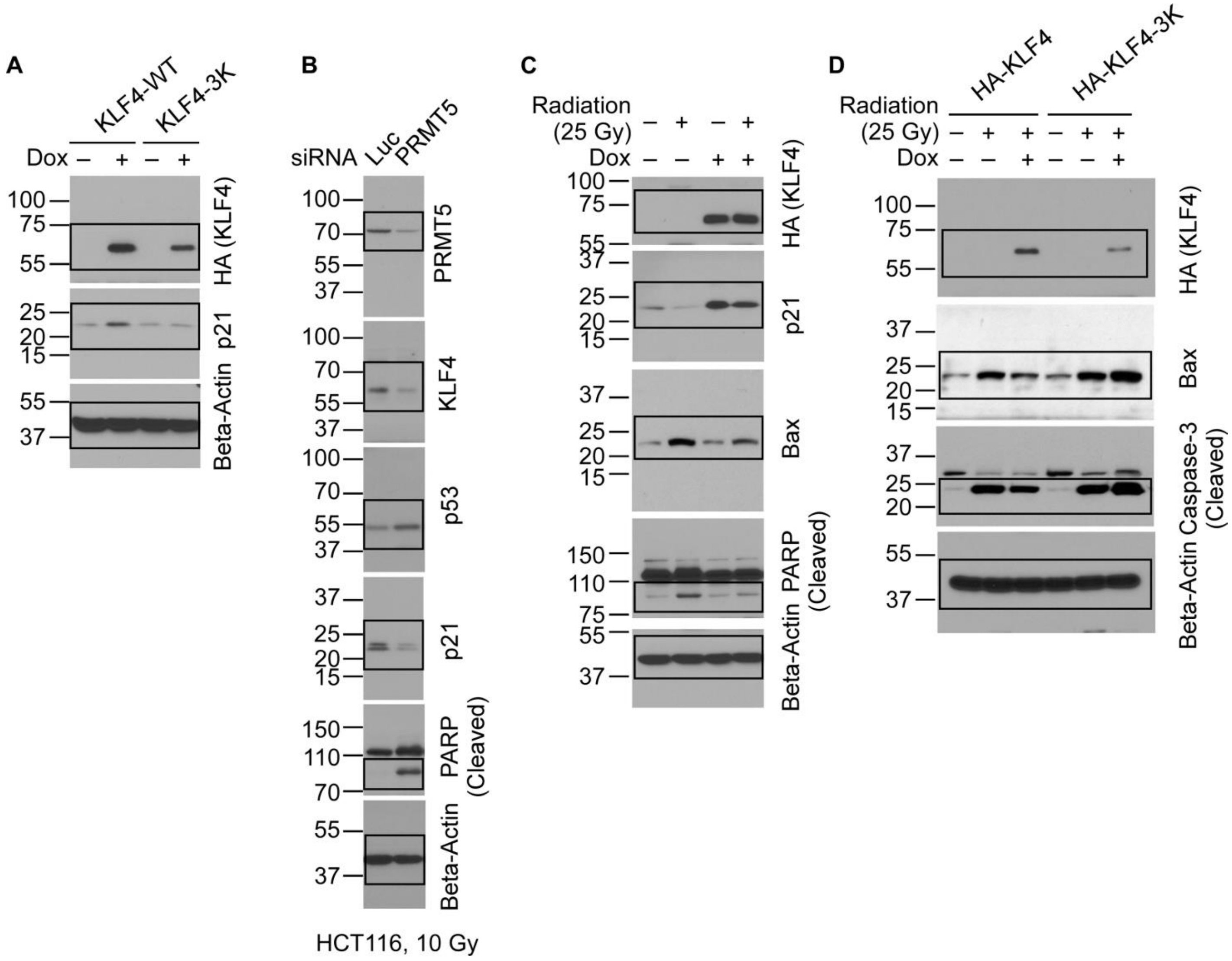

Supplementary Figure 15. Full Scans of Western blots

Supplementary Figure 7

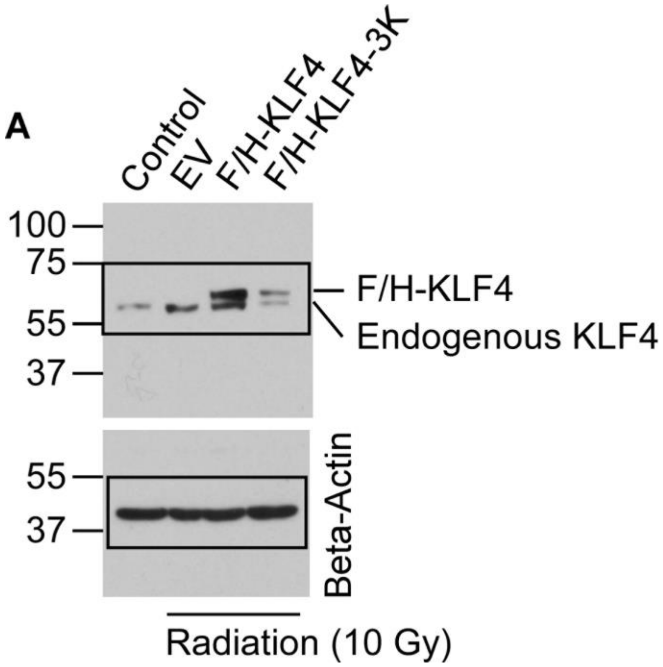

Supplementary Figure 16. Full Scans of Western blots

Supplementary Figure 8

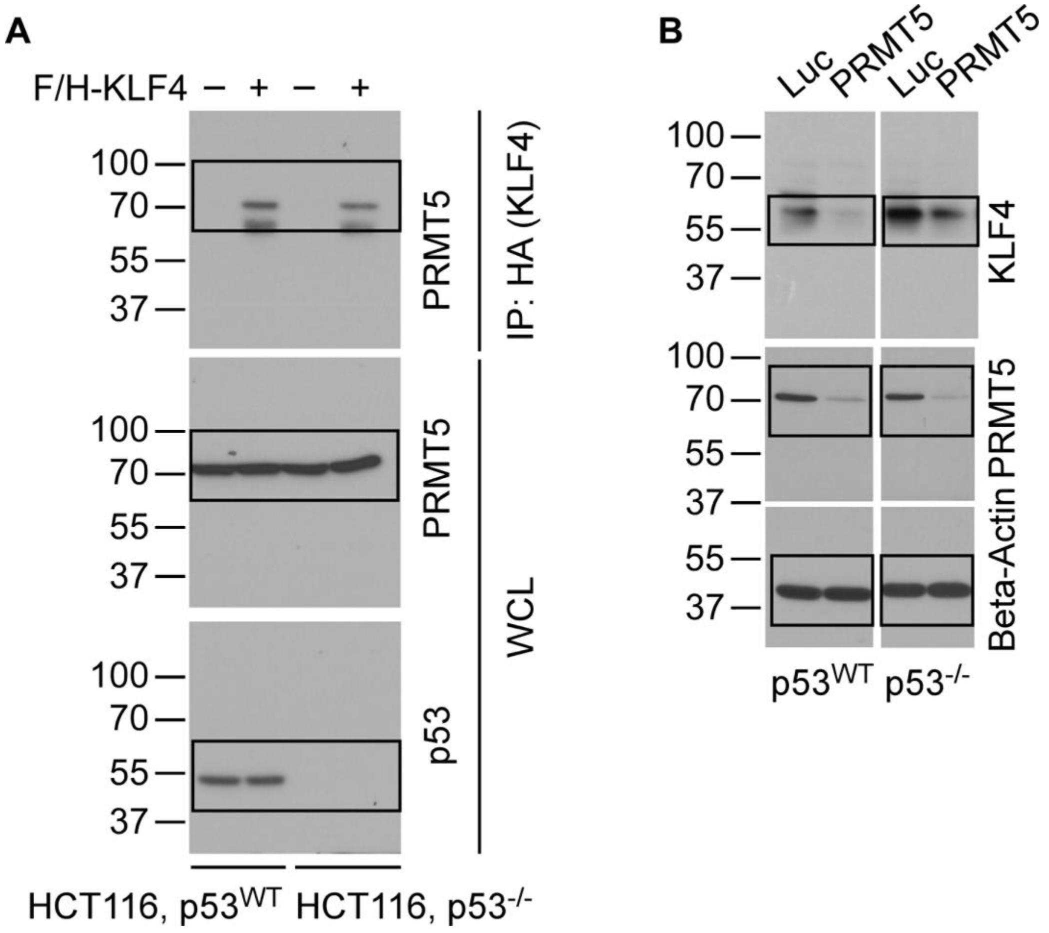

Supplementary Figure 17. Full Scans of Western blots

Supplementary Figure 9

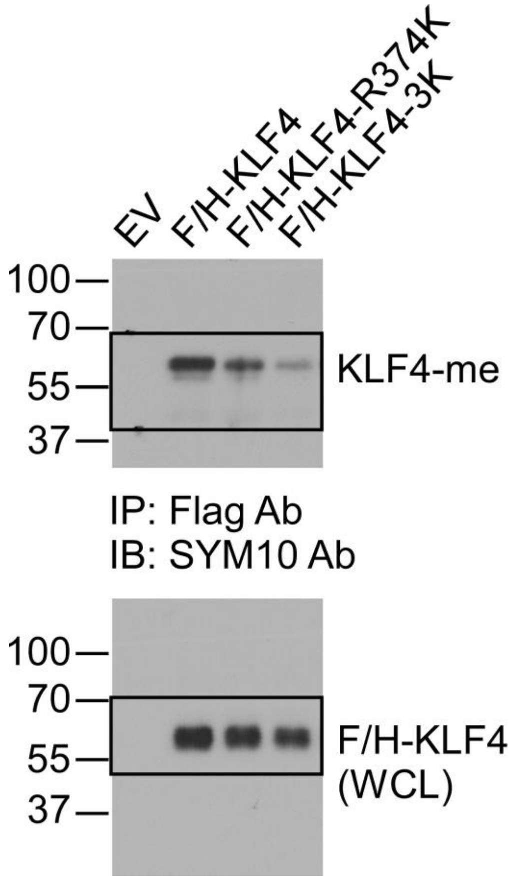

Supplementary Figure 18. Full Scans of Western blots

SUPPLEMENTARY TABLES

Supplementary Table 1. Protocol for generating the first PRMT5-KLF4 complex

	**Type of Simulation**	**Aim**	**Steered/ constrained atoms**	**Direction**	**Duration (ns)**
**1**	sMD[(a)]	Steering KLF4 into the PRMT5 dimer	K382-H411 of KLF4	Towards N482-E489 of PRMT5	2
**2**	cMD	Relaxing conformers	None	None	22
**3**	sMD[(b)]	Bringing R377 (KLF4) to the entrance of the narrow tunnel formed by PRMT5 residues L312, F327 and W579, and subsequently steering R377 into the active site through this tunnel.	R377 of KLF4	Visually updated as simulation proceeded	16
**4**	cMD	Relax structures	None		10
**5**	sMD	Steering oppositely charged residues on binding regions towards each other.	K382 and R383 of KLF4	Towards E483 of PRMT5	4
**6**	cMD	Allow structure the readjust around these coordinates.	R377, K382, and R383 of KLF4 (constrained atoms)		6
**7**	sMD	Steering R377 out and R374 into the active site through the tunnel	R374, R377, K382 and R383 of KLF4	Interactively updated as simulation proceeded	6
**8**	cMD	Allowing the complex to rearrange in the neighborhood of this bound conformer	R374 and R383 of KLF4. E435 and E444, and E483 of PRMT5.		20
					15
**Total**					**101 ns**

[a]*In the sMD simulations the steering direction is defined as the vector connecting the center of mass of the steered atoms and those of the fixed atoms.*
[b]*Fixed atoms were changed to change the steering directions.*

Supplementary Table 2. Protocol for the series of MD simulations performed to refine the PRMT5-KLF4 binding mode

	**Type of Simulation**	**Aim**	**TMD atoms**	**Additional Constraints**	**Duration (ns)**
1	tMD	Arrange KLF4 to attain the binding mode of MD1 (obtained in step 4 of the procedure)	C□ atoms P370-A385, T386-C389 (ββ), and G395-K400(ββ) of KLF4 (new coordinates). C□ atoms of PRMT5 (current coordinates).	Distance between C□ atoms of KLF4 residues T386, H387, T388, K400, P416, S402, L404, R409, and residues Y398, T397, K396, T412, A429, R409, L408, S402, respectively.	6
2	cMD	Allow KLF4 readjust in this binding mode.		Positions of all C□ atoms.	4
3	cMD	Allow KLF4 readjust in this binding mode.		Positions of all C□ atoms. k=1 kcal/mol /Å^2	4
4	cMD	Refine binding mode			230x2= 460

Supplementary Table 3. KLF4 Expression and Clinicopathological Features of IBC

Clinicopathological Features	Cases	KLF4 expression		*rs*	*P*-value*
		-	+		
Age				0.085	0.146
≤55	145	70(48)	75(52)		
＞55	59	23(39)	36(61)		
Histological grade				0.204	0.011
I	35	23(66)	12(34)		
II	123	55(45)	68(58)		
III	46	15(33)	31(67)		
LN metastasis				0.083	0.236
N0	103	52(51)	51(49)		
N1	96	39(41)	57(59)		
N≥2	5	2(40)	3(60)		
pTNM Stage				0.086	0.086
Tis,T1	16	12(75)	4(25)		
T2	89	39(44)	50(56)		
T3	72	29(40)	43(60)		
T4	27	13(48)	14(52)		
Estrogen receptor				0.022	0.44
-	147	68(46)	73(54)		
+	57	23(44)	32(56)		
Progesterone receptor				0.004	0.959
-	151	69(46)	82(54)		
+	53	24(45)	29(55)		
C-erbB-2				-0.248	0.00032
-	113	39(35)	74(65)		
+	91	54(59)	37(41)		
EGFR				0.070	0.203
-	158	75(47)	83(53)		
+	46	18(39)	28(61)		
Ki67				-0.105	0.133
-	134	56(42)	78(58)		
+	70	37(53)	33(47)		

**P*-values were calculated by Spearman's Rank-Correlation test (n=204).

Supplementary Table 4. PRMT5 Expression and Clinicopathological Features of IBC

Clinicopathological Features	Cases	PRMT5 expression -	+	r_s	*P*-value*
Age				-0.007	0.522
≤55	145	53(37)	92(63)		
>55	59	22(37)	37(63)		
Histological grade				0.049	0.031
I	35	10(29)	25(71)		
II	123	54(44)	69(56)		
III	46	11(24)	35(76)		
LN metastasis				-0.156	0.142
N0	103	31(30)	72(70)		
N1	96	41(43)	55(57)		
N≥2	5	3(60)	2(40)		
pTNM Stage				-0.007	0.685
Tis,T1	16	6(37)	10(63)		
T2	89	34(38)	55(62)		
T3	72	23(32)	49(68)		
T4	27	12(44)	15(56)		
Estrogen receptor				0.044	0.527
-	147	56(38)	91(33)		
+	57	19(33)	38(63)		
Progesterone receptor				0.081	0.162
-	151	59(39)	92(61)		
+	53	16(30)	37(70)		
C-erbB-2				-0.400	9.8×10^{-9}
-	113	22(19)	91(81)		
+	91	53(58)	38(42)		
EGFR				-0.099	0.107
-	158	54(34)	104(66)		
+	46	21(46)	25(54)		
Ki67				0.123	0.054
-	134	55(41)	79(59)		
+	70	20(29)	50(71)		

**P*-values were calculated by Spearman's Rank-Correlation test (n=204).

SUPPLEMENTARY DISCUSSION

The PRMT5-KLF4 complex from the second run

R374 of KLF4 is inside the active site of PRMT5 and forming salt bridges with residue E444. As it was the case for the first run (discussed in main text), a very strong interaction network exists comprising residues R381, K382, R383 of KLF4 and residues E483, E489 of PRMT5, as shown in Supplementary Figure 4: Salt bridges are formed between E483 (PRMT5) and R381 (KLF4). PRMT5 (first protomer, shown in grey color in the figures) residue E489 is forming a salt bridge with the KLF4 residues K382. Backbone carbonyl group of A486 (PRMT5) is forming hydrogen bonds with the backbone amine groups of K382 and R383.

In addition to this strong and localized interaction network, also other significant interactions were observed at the experimentally predicted binding region of PRMT5, as it was the case for the first run. Residue E489 of the second PRMT5 protomer (shown in *lime* in the figures) is forming a salt bridge with KLF4 residue K396. H403 on KLF4 is interacting with E483 on the second PRMT5 protomer. These interactions are further complemented with interactions between backbone amines and carbonyls such as backbone carbonyl of R488 (PRMT5) and backbone amines of T399 and K400.

SUPPLEMENTARY METHODS

Modeling the N-terminal region of the unbound KLF4

In order to model KLF4 in its unbound form, first the DNA was removed from its crystal structure (PDB ID: 2WBS [1]) (Supplementary Figure 2A). Subsequently, the missing region of our interest (residues 370-383; PKPKRGRRSWPRKR) was modeled using Coot [2] (Crystallographic Object-Oriented Toolkit) as an unstructured N-terminal "tail", supported by the unstructured regions next to the first zinc fingers in the NMR/solution structures of KLF3 [3], KLF10 (PDB ID:2EPA, unpublished), KLF15 (PDB ID:2ENT, unpublished) and Sp1 [4], as well as the dispersed electron distributions observed at the N-terminal end of the X-ray structure resolved for KLF4 (PDB ID: 2WBS). In addition, the sequence analysis performed by Shuetz et al. predicts the N-terminus of KLF4 to be unfolded [1]. We performed two independent MD simulations of KLF4, 120ns and 100ns in length, in which the newly added N-terminal "tail" showed no tendency to get structured. Significantly, the simulations revealed that the regions connecting consequent zinc finger domains act like hinges, making the structure highly mobile. The mobility, as will be discussed in the subsequent sections, gives KLF4 the required flexibility to enter and bind to the PRMT5 dimer.

Modeling PRMT5 Structure

C. elegans PRMT5 was shown to exists as a homodimer both in the solution and crystal structure [5]. The *human* PRMT5, on the other hand, binds to MEP50 and forms a hetero-octameric complex (4 PRMT5s and 4 MEP50s) [6]. The residues and interactions across the PRMT5 dimer interface are preserved between the *C.elegans* PRMT5 dimer and the human PRMT5: MEP50 hetero-octamer, suggesting that the structure of the PRMT5 dimer is conserved across species and is likely to be adopted by human PRMT5 in the absence of MEP50 [6]. We, therefore, selected the *human* dimer from the hetero-octamer (PDB ID:4GQB)

for structural and dynamical analysis. S-adenosylmethionine analog A9145[11] and the substrate peptide derived from Histone H4 were removed from the active site. The apo PRMT5 homodimer was subsequently simulated for 41ns in the presence of explicit solvent, which showed that the apo PRMT5 dimer is quite stable; having only a RMSD of 2.4Å from its crystal backbone coordinates at the end of the simulation.

Details of MD simulations

Structures were solvated in a box having at least 10Å of water in each direction from the exposed atoms. Ions were added (150 mM NaCl) to neutralize the system. A cutoff distance of 12Å was adopted for van der Waals interactions, with a switching function that starts at 10Å and reaches zero at 12Å. Long-range electrostatic forces were computed using the particle-mesh Ewald method. The equilibrated structures were generated by two energy minimization-equilibration procedures: the first at constant temperature (T=310 K) and pressure (P=1 atm) (NPT ensemble) with the C^{α} atoms held fixed; and the following at constant temperature and volume (canonical ensemble). A damping coefficient of 0.5 ps^{-1} was used to maintain isothermal conditions, and NPT simulations with Langevin Nosé-Hoover method were performed to maintain the pressure constant. Time steps of 1fs were used. The size of the simulated systems varied from 41,609 (KLF4 alone) to 327,992 atoms (KLF4 and PRMT5 dimer). For steered MD (sMD) simulations a spring constant of k=5 kcal/mol /$Å^2$ was applied. A range of steering velocities, 2-17 Å/ns, were used depending on the number of surrounding residues in contact: When steered through an environment where many contacts exist low steering velocities were applied. In the targeted MD (tMD) simulations an elastic constant of k=2000 kcal/mol /$Å^2$ for TMD forces was used. For harmonic constraints a force constant of

k=2 kcal/mol /Å^2 was applied. For atom-atom distance constraints, on the other hand, a force constant value of k=50 kcal/mol /Å^2 was used. If not indicated otherwise in the tables the above indicated force constants were used in all simulations.

Modeling KLF4-PRMT5 binding

Steering KLF4 into the cavity formed by the PRMT5 dimer: KLF4 has to enter into the large cavity formed by the PRMT5 dimer (Supplementary Figure 2D) in order to access the experimentally predicted binding region and the active site. For this purpose, first KLF4 was placed 10Å away from the PRMT5 dimer, in such an orientation that the experimentally predicted binding regions of KLF4 and PRMT5 were facing each other. Subsequently, KLF4 was steered into the PRMT5 dimer by applying steering forces to its first zinc finger (Supplementary Table 1); which is the zinc finger that spans a part of the experimentally predicted KLF4 binding region (Supplementary Figure 2B-C). Once inside the PRMT5 crevice, conventional MD (cMD) simulations were performed to allow KLF4 to relax and rearrange in its new location.

Steering arginine R377 into the active site: In order to get methylated, arginines 374, 376 and 377 need to first enter the active site of PRMT5 through the narrow tunnel formed by the PRMT5 residues L312, F327 and W579 (Supplementary Figure 2E-F). Among these arginines, R377 is the spatially and also sequentially closest one to the experimentally predicted PRMT5 binding region on KLF4 (See Supplementary Figure 2B). Hence, R377 binding to glutamates 435 and/or 444 in the active site of PRMT5 will spatially restrain (quasi pin down) KLF4 and, by doing so, strongly limit available binding modes. This spatial constraint will in turn drastically

narrow down the search for the most probable binding mode. For this purpose, among arginines 374, 376 and 377, first R377 was steered into the active site of PRMT5 by a set of consecutive sMD and cMD simulations, totaling 26ns in length (Simulations 3-4 in Supplementary Table 1). During these simulations the directions of the steering forces were interactively updated so that R377 followed a pathway passing through the narrow tunnel, as suggested by Antonysamy et al. [6]. The direction of steering forces was changed mainly by updating the selection of fixed atoms, which are the atoms towards which the selected "steered" atoms are pulled.

Forming an initial bound conformation based on the surface chemical properties: The PRMT5 binding region on KLF4, KRTATHT, despite being only 7 residues long, comprises two positively charged and 4 polar residues; making it *potentially* highly interactive. If R377 is bound to E435 and/or E444, then the PRMT5 residue E483 is the only accessible oppositely charged residue to the positively charged KLF4 residues K382 and R383 on the experimental predicted binding region (Supplementary Figure 2G). We will use this "spatial constraint" to generate a starting conformation for the search of the most probable KLF4-PRMT5 binding mode; quasi an initial guess. For this purpose KLF4 residues K382 and R383 were steered towards E483 on PRMT5, and constrained at this position so that KLF4 can rearrange and form new interactions accordingly (See SupplementaryTable 1 for details). While K382 and R383 are kept further constrained at this location (aiming to optimize the interactions in this binding mode) R377 was pulled out and R374, which was picked up the most in our mass spec experiments (see Figure 2E), was steered into the active site through the narrow tunnel. R374, R383 on KLF4, and E435 444 and 483 on PRMT5 were restrained for an additional 20ns of MD simulations to allow new interactions to form and the KLF4 structure to optimize around

this binding mode. As a final step all restrains were removed and the system was simulated for an additional 15ns.

Supplementary Table 1 describes the protocol for generating a first binding pause, step 1, 2 and 3 (overall simulation duration of 101 ns), which has been subjected to further refinements using MD simulations of ~1.5 microseconds. A movie showing the protocol described in Supplementary Table 1 is provided as Supplementary Movie 1.

Refining the binding mode: KLF4 is filling up much of the crevice formed by the PRMT5 dimer by having at least the first zinc finger domain inside the crevice. This, in turn, makes it difficult to sample different binding modes in feasible MD simulations lengths; the KLF4-PRMT5 dimer complex inside the water box has more than 200,000 atoms which strongly limits accessible simulations lengths. To circumvent this sampling issue we removed the KLF4 structure after residue T388 (removing residues C399-F470) leaving only residues P370-T388 of KLF4 and, hence, no zinc finger inside the PRMT5 crevice. As a result, the degrees of freedom of the *region of interest* in KLF4 increased drastically. In addition the size of the simulated biological system reduced by almost half; increasing accessible time scales radically. We performed the same procedure to 3 different KLF4-PRMT5 conformers; each selected from the last 1ns of the previous step, and simulated each run for 465 ns, 280 ns and 270 ns (MD1, MD2 and MD3, respectively). As can be inferred by their root mean sequare deviations (RMSD) from their initial binding location/conformation, as shown in Supplementary Figure 2H, KLF4 converged to a specific binding mode in each of these simulations. The resulting binding modes are depicted in Supplementary Figure 2I**.** Strikingly, in all simulations the salt bridge E444-R374 between PRMT5-KLF4 was conserved. Simulations showed that the experimentally predicted

binding region on KLF4 has high affinity to the dimerization domain of PRMT5, which is located inside our experimentally predicted binding region on PRMT5.

Rearranging KLF4 to mimic the most meaningful binding modes: Among the three binding modes obtained in the previous step, MD1 (simulated for 465 ns) resulted in the highest number of interactions between the KLF4 residues K382-T388 and the PRMT5 residues P465-H510; the experimentally predicted binding regions. Now, KLF4 coordinates in the KLF4-PRMT5 complex (obtained in step 3 based on surface chemical properties) need to be adjusted to mimic the resulting binding mode of MD1. For this aim first tMD simulations followed by cMD simulations were performed. For the tMD simulations coordinates of residues P370-A385 were directly taken from the final conformer of MD1 (after aligning conformers with respect to their PRMT5s, Supplementary Figure 3) Since the residue stretch T386-T388 of our experimentally predicted binding region is a part of the well defined ββ structure of the first zinc finger (which we would like to conserve) we first aligned this ββ structure to the end conformer of MD1 with respect the backbone atoms I386-L388 (Supplementary Figure 3). The resulting coordinates of the ββ structure were added to our set of target coordinates. In addition internal constraints were introduced to the ββ structure to preserve its structure (See Supplementary Table 3). tMD simulations were performed followed by restrained simulations so that the system can adjust in these new targeted coordinates. Finally, two independent sets of conventional MD simulations, 230ns each, were performed to refine the binding mode. The procedure is schematically shown in Supplementary Figure 3.

SUPPLEMENTARY REFERENCES

1. Schuetz, A. *et al.* The structure of the Klf4 DNA-binding domain links to self-renewal and macrophage differentiation. *Cellular and molecular life sciences : CMLS* **68**, 3121-3131 (2011).
2. Emsley, P., Lohkamp, B., Scott, W.G. & Cowtan, K. Features and development of Coot. *Acta Crystallogr D Biol Crystallogr* **66**, 486-501 (2010).
3. Simpson, R.J. *et al.* CCHX zinc finger derivatives retain the ability to bind Zn(II) and mediate protein-DNA interactions. *J Biol Chem* **278**, 28011-28018 (2003).
4. Oka, S. *et al.* NMR structure of transcription factor Sp1 DNA binding domain. *Biochemistry* **43**, 16027-16035 (2004).
5. Sun, L. *et al.* Structural insights into protein arginine symmetric dimethylation by PRMT5. *Proceedings of the National Academy of Sciences of the United States of America* **108**, 20538-20543 (2011).
6. Antonysamy, S. *et al.* Crystal structure of the human PRMT5:MEP50 complex. *Proceedings of the National Academy of Sciences of the United States of America* **109**, 17960-17965 (2012).
